# Influence of arylene diimide linker aromaticity on the electrochemical performance of redox-active metal–organic framework cathodes for lithium-ion storage

**DOI:** 10.1039/d6ta05048d

**Published:** 2026-10-12

**Authors:** Catarina Ribeiro, Amol Kumar, Jonathan Caroni, Gioele García-Pinar, A. Nuno Guerreiro, João Rocha, M. Helena Braga, Joaquín Calbo, Sascha Ott, Manuel Souto

**Affiliations:** a Department of Chemistry, CICECO-Aveiro Institute of Materials, University of Aveiro 3810-393 Aveiro Portugal; b Department of Chemistry - Ångström Laboratory, Uppsala University 75120 Uppsala Sweden sascha.ott@kemi.uu.se; c Centro Singular de Investigación en Química Biolóxica e Materiais Moleculares (CiQUS), Departamento de Química-Física, Universidade de Santiago de Compostela 15782 Santiago de Compostela Spain manuel.souto.salom@usc.es; d Instituto de Ciencia Molecular (ICMol), Universidad de Valencia 46980 Paterna Spain joaquin.calbo@uv.es; e MatER, Faculty of Engineering, University of Porto 4200-465 Porto Portugal; f LAETA, Institute of Science and Innovation in Mechanical and Industrial Engineering 4200-465 Porto Portugal; g Oportunius, Galician Innovation Agency (GAIN) 15702 Santiago de Compostela Spain

## Abstract

Organic electrode materials represent an attractive alternative to inorganic cathodes because they are based on abundant elements and offer tunable electrochemical performance through molecular design. Among them, metal–organic frameworks (MOFs) incorporating redox-active organic linkers have attracted considerable attention as electrode materials for metal-ion batteries. Herein, a series of isoreticular redox-active MOFs based on pyromellitic diimide (PMDI), naphthalene diimide (NDI), and perylene diimide (PDI) linkers were investigated as cathode materials for lithium-ion storage to elucidate the influence of arylene diimide aromaticity on electrochemical performance. Among the Zn-XDI (XDI = PMDI, NDI, PDI) MOFs, Zn-PDI and Zn-NDI exhibited excellent cycling stability, retaining 98% and 87% of their initial capacities after 500 cycles at 2C, respectively, whereas Zn-PMDI showed poor capacity retention, highlighting the importance of the aromatic core in stabilizing reduced states. Post-mortem X-ray photoelectron spectroscopy (XPS), powder X-ray diffraction and Raman studies confirmed the preservation of the framework structure of Zn-PDI and Zn-NDI after extended cycling. Density functional theory (DFT) calculations revealed that increasing the aromatic core size in the Zn-XDI series stabilizes the reduced states, lowers the LUMO energy, enhances electron affinity, and facilitates Li^+^ uptake through the carbonyl oxygen atoms. These findings establish a direct relationship between linker aromaticity and electrochemical performance and provide molecular-level design principles for the development of high-performance redox-active MOF cathodes for sustainable energy storage.

## Abstract 2

Isoreticular Zn-XDI MOFs with PMDI, NDI and PDI linkers reveal that increasing aromaticity stabilizes reduced states and enhances Li-ion storage. Zn-PDI and Zn-NDI show excellent cycling stability, establishing aromaticity as a key design parameter.

## Graphical abstract

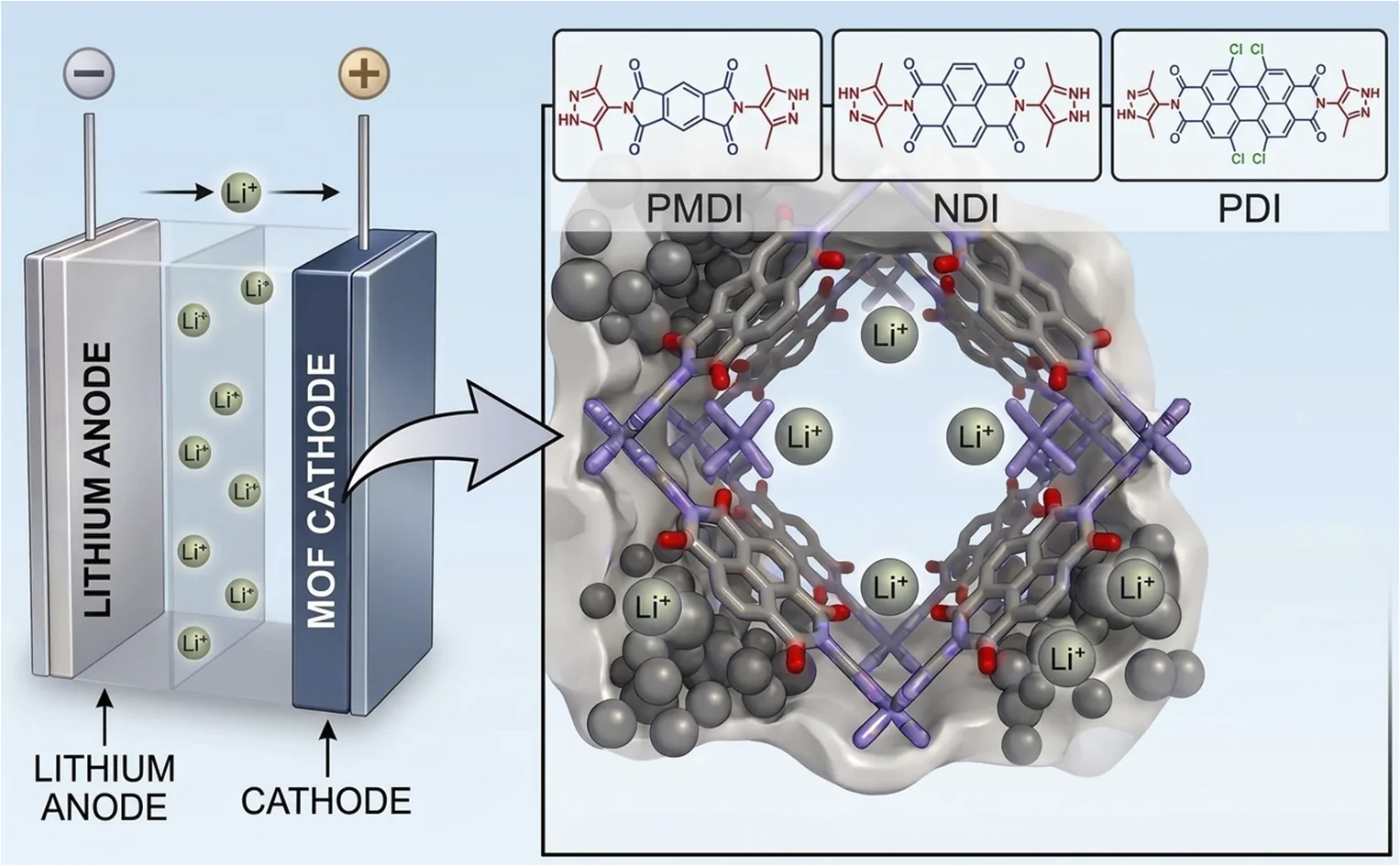

## Introduction

Electrical energy storage devices play a crucial role in the transition away from conventional fossil fuels, as they efficiently store electricity generated from renewable sources. Lithium-ion batteries are widely regarded as one of the most promising electrochemical energy storage technologies, owing to their high energy density, low self-discharge rate, long cycle life, and widespread application in portable electronics and the electric vehicle sector.^1–3^ However, there is a growing need to replace conventional transition metal oxide-based cathodes to overcome intrinsic limitations in specific capacity and to reduce dependence on the mining of critical raw materials, particularly cobalt.^4,5^ Organic electrode materials (OEMs) have emerged as a promising alternative to inorganic cathodes, as they are composed of abundant elements and their electrochemical performance can be adjusted through molecular design.^6–10^ Nevertheless, a major challenge associated with small organic molecules is their solubility in conventional battery electrolytes, which often results in rapid capacity fading. In this regard, an effective strategy to overcome this issue involves the polymerization of redox-active organic molecules to enhance insolubility and cycling stability, incorporating them into conventional redox polymers,^11^ covalent organic frameworks (COFs),^12–14^ and metal–organic frameworks (MOFs).^15–17^

Electroactive MOFs based on redox-active organic linkers have emerged as promising materials for energy storage applications.^18–21^ For example, MOFs incorporating anthraquinone,^22–24^ benzoquinone,^25,26^ tetrathiafulvalene,^27–29^ and naphthalene diimide (NDI)^30,31^ redox-active moieties have been investigated as electrode materials for various metal-ion batteries. In the case of an NDI-based MOF, a pyridyl-functionalized NDI linker was combined with Cd(ii) ions to construct a redox-active framework, which was investigated as a cathode material for lithium-ion storage but showed limited electrochemical performance and stability.^30^

Organic molecules^32^ and conventional linear polymers^33–39^ based on arylene diimides such as NDI, pyromellitic diimide (PMDI), and perylene diimide (PDI) have been widely investigated as OEMs for batteries. A recent study demonstrated that modulating the aromatic ring size of arylene diimide molecules can tune their electrochemical properties.^32^ In contrast, only a very limited number of studies have explored arylene diimide linkers such as NDI or PDI within MOF architectures as battery cathodes, and a systematic evaluation of the influence of the aromatic core within an isostructural MOF series is still lacking.

Herein, we report a systematic investigation of the electrochemical performance and charge-storage mechanism of an isostructural series of redox-active arylene diimide-based Zn-XDI MOFs (XDI = PMDI, NDI, PDI) (Fig. 1) as cathode materials for lithium-ion storage. This design enables us to assess the influence of the aromatic arylene diimide moieties on the electrochemical properties. While Zn-NDI and Zn-PDI show good capacity retention (87 and 98%, respectively) after 500 cycles at 2C, Zn-PMDI exhibited poor electrochemical performance under the same conditions. Rate capability measurements reveal superior high-rate performance for Zn-PDI, which is attributed to its predominantly pseudocapacitive charge storage behavior. In addition, post-mortem analysis by *ex situ* X-ray photoelectron spectroscopy (XPS), powder X-ray diffraction (PXRD) and Raman spectroscopy demonstrates the high electrochemical stability of Zn-NDI and Zn-PDI MOFs after 1000 cycles. Quantum chemical calculations further demonstrate that the aromatic ring size plays a key role in stabilizing the intermediate reduced species formed during the lithiation/delithiation processes. Understanding these structure–performance correlations is essential for the molecular design of redox-active MOFs as high-performance electrode materials for metal-ion batteries.

**Fig. 1:**
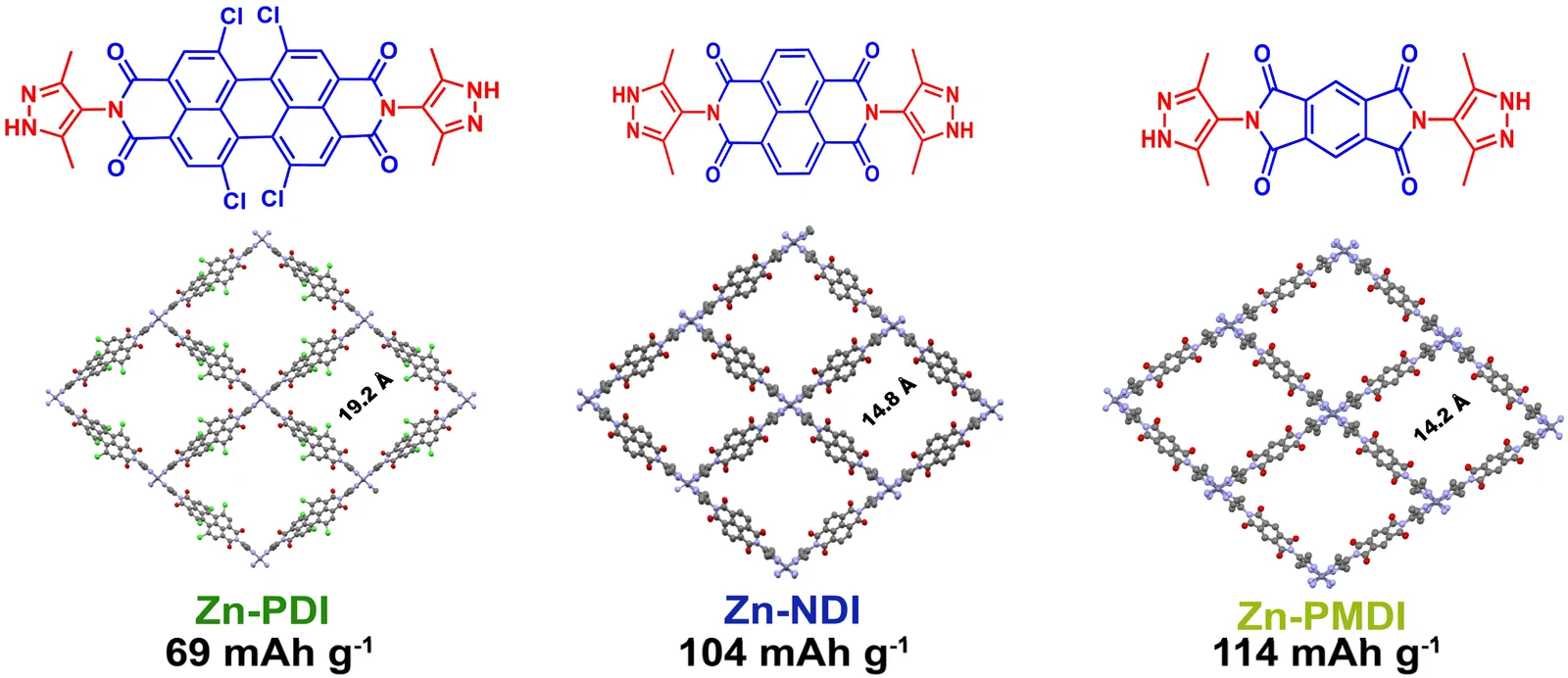
Representation of the arylene diimide (XDI) linkers and the corresponding Zn-XDI MOFs (Zn-PDI, Zn-NDI, and Zn-PMDI), along with their theoretical specific capacities.

## Results and discussion

### Synthesis and characterization of Zn-XDI MOFs

The isoreticular and highly stable Zn-XDI frameworks were synthesized following a previously reported solvothermal procedure.^40^ Briefly, the pyrazole-XDI linker (0.10 mmol) and Zn(NO_3_)_2_·6H_2_O (0.11 mmol) were dissolved in DMF (10 mL), sonicated to obtain a homogeneous solution, and degassed with Argon for 10 min. The reaction vials were sealed and heated at 135 °C for 4.5 h. The resulting microcrystalline powders were isolated by centrifugation, washed with DMF, and solvent-exchanged with methanol (see the SI for details). The crystallinity and structural integrity of the obtained MOFs were confirmed by powder X-ray diffraction (PXRD, Fig. 2), which showed reflections consistent with the expected framework structures based on the reported structural models.^40–42^ Pawley refinements further confirmed the agreement between the experimental PXRD data and the patterns simulated from these models (Fig. S1–S3). Additional characterization, including permanent porosity measurements, pore size distribution, BET surface area analysis, scanning electron microscopy (SEM), and thermogravimetric analysis (TGA), is provided in the SI (Fig. S4–S8).

**Fig. 2:**
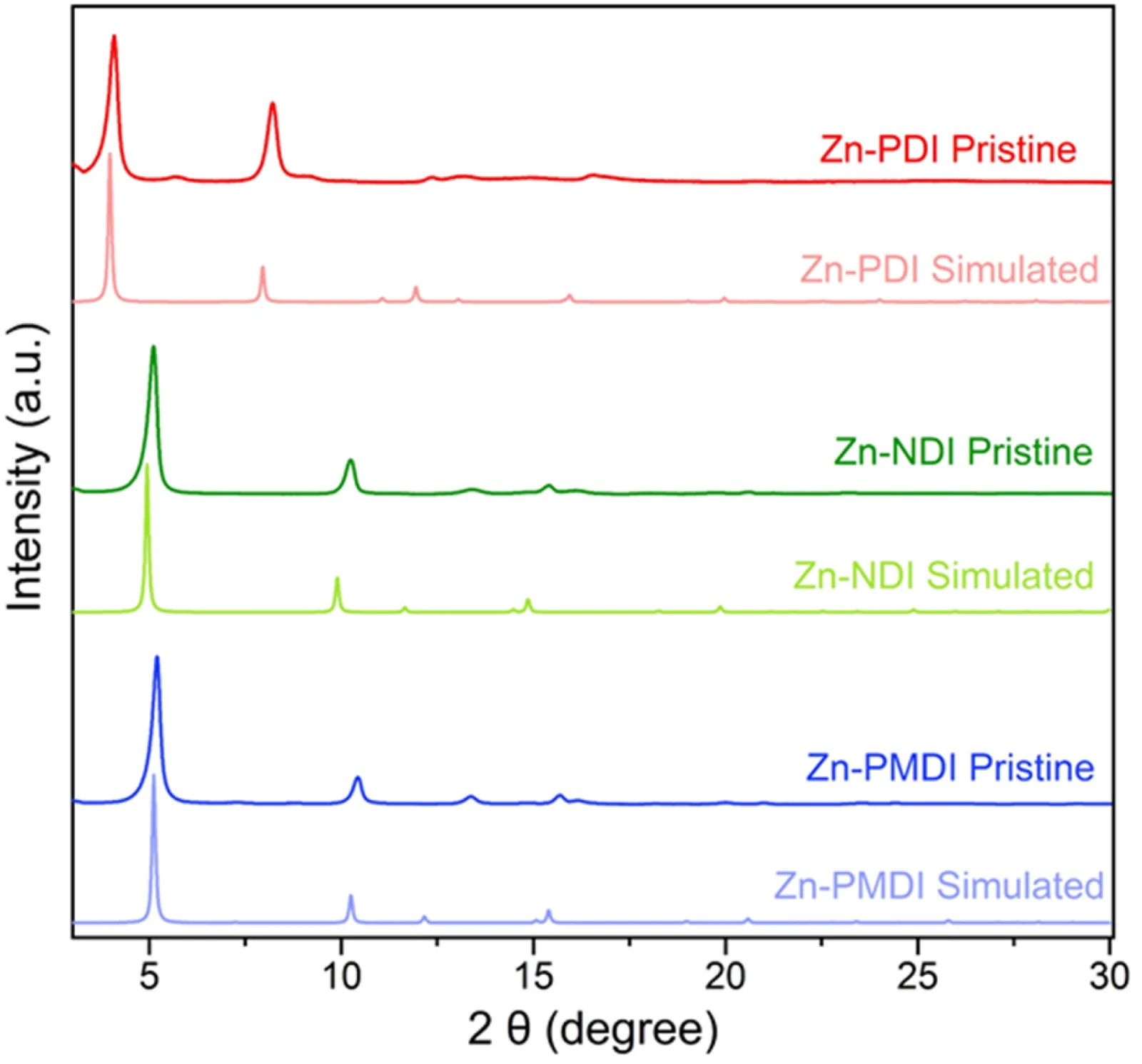
Simulated and experimental PXRD patterns of the isoreticular Zn-XDI MOF series.

### Electrochemical performance

The electrochemical performance of Zn-XDI MOFs was evaluated as cathode materials for lithium-ion storage. The working electrodes were prepared by mixing the active material (Zn-XDI MOFs), conductive carbon black, and poly(vinylidene fluoride) (PVDF, binder) in a mass ratio of 50 : 40 : 10. The mass loading of the active material ranged from 0.4 to 0.9 mg cm^−2^. The carbon black content was selected considering the relatively low intrinsic conductivity of Zn-XDI MOFs,^43^ together with previous studies demonstrating the importance of conductive additives for achieving efficient electronic transport in MOF-based battery electrodes.^22–24^ To assess the influence of the conductive additive content, electrodes with a 60 : 30 : 10 mass ratio of active material/conductive carbon/PVDF were also evaluated. As shown in Fig. S9, both Zn-NDI and Zn-PDI retain their electrochemical response over 500 cycles at 2C, although lower initial specific capacities are observed. These results support a significant contribution of the Zn-XDI frameworks to the measured capacity. Electrochemical measurements were conducted in coin-type half-cells using Li metal as both the reference and counter electrodes within a potential window of 1.4–3.5 V. All measurements were performed using 1 M lithium bis(trifluoromethanesulfonyl)imide (LiTFSI) dissolved in a 1 : 1 (v/v) mixture of 1,3-dioxolane (DOL) and dimethoxyethane (DME) as the electrolyte. Electrochemical impedance spectroscopy (EIS) measurements of Zn-NDI and Zn-PDI were carried out prior to cycling at room temperature over a frequency range of 1 MHz to 0.1 Hz with a sinusoidal amplitude of 10 mV (Fig. S10). The EIS results indicate that the Zn-PDI electrode exhibits a lower charge-transfer resistance than the Zn-NDI MOF electrode, which can be attributed to the enhanced surface conductivity of the Zn-PDI material.

The cycling stability of Zn-XDI MOFs as cathodes for lithium-ion storage was first evaluated at a current density of 2C within a potential window of 1.4–3.5 V *vs.* Li/Li^+^ (Fig. 3). The theoretical capacities of Zn-PDI, Zn-NDI, and Zn-PMDI are calculated to be 69, 104, and 114 mAh g^−1^, respectively (Fig. 1). The initial discharge capacities were 49, 116, and 13 mAh g^−1^ for Zn-PDI, Zn-NDI, and Zn-PMDI, respectively. These capacities decrease during the first cycles before reaching a stable capacity retention upon progressive electrochemical activation. After 500 cycles, the capacity retention values were 98% and 87% for Zn-PDI and Zn-NDI, respectively. In contrast, Zn-PMDI exhibited poor capacity retention (36%) after 500 cycles. A plausible explanation for this behavior is solubility-related degradation and poorer stabilization of the charged species when using PMDI moieties^32^ (see quantum-chemical calculations section). This lack of stability during repeated charge–discharge cycling is further supported by cyclic voltammetry (CV) measurements of the Zn-PMDI MOF in 1 M LiTFSI in DOL : DME, recorded at a scan rate of 1.0 mV s^−1^ over the potential range of 1.4–3.5 V (Fig. S11). To investigate the contrasting early-stage behavior for Zn-NDI and Zn-PDI, EIS measurements were performed during cycling. Zn-NDI exhibited an initial increase in impedance followed by a decrease, whereas Zn-PDI showed a continuous impedance decrease consistent with progressive electrochemical activation (Fig. S12). To further investigate electrolyte compatibility, the Zn-XDI MOFs were also evaluated in 1 M lithium hexafluorophosphate (LiPF_6_) dissolved in a 1 : 1 (v/v) mixture of ethylene carbonate (EC) and diethyl carbonate (DEC) (Fig. S13). This electrolyte resulted in an overall inferior electrochemical performance and stability compared to 1 M LiTFSI in DOL/DME, as observed for other related systems.^44^

**Fig. 3:**
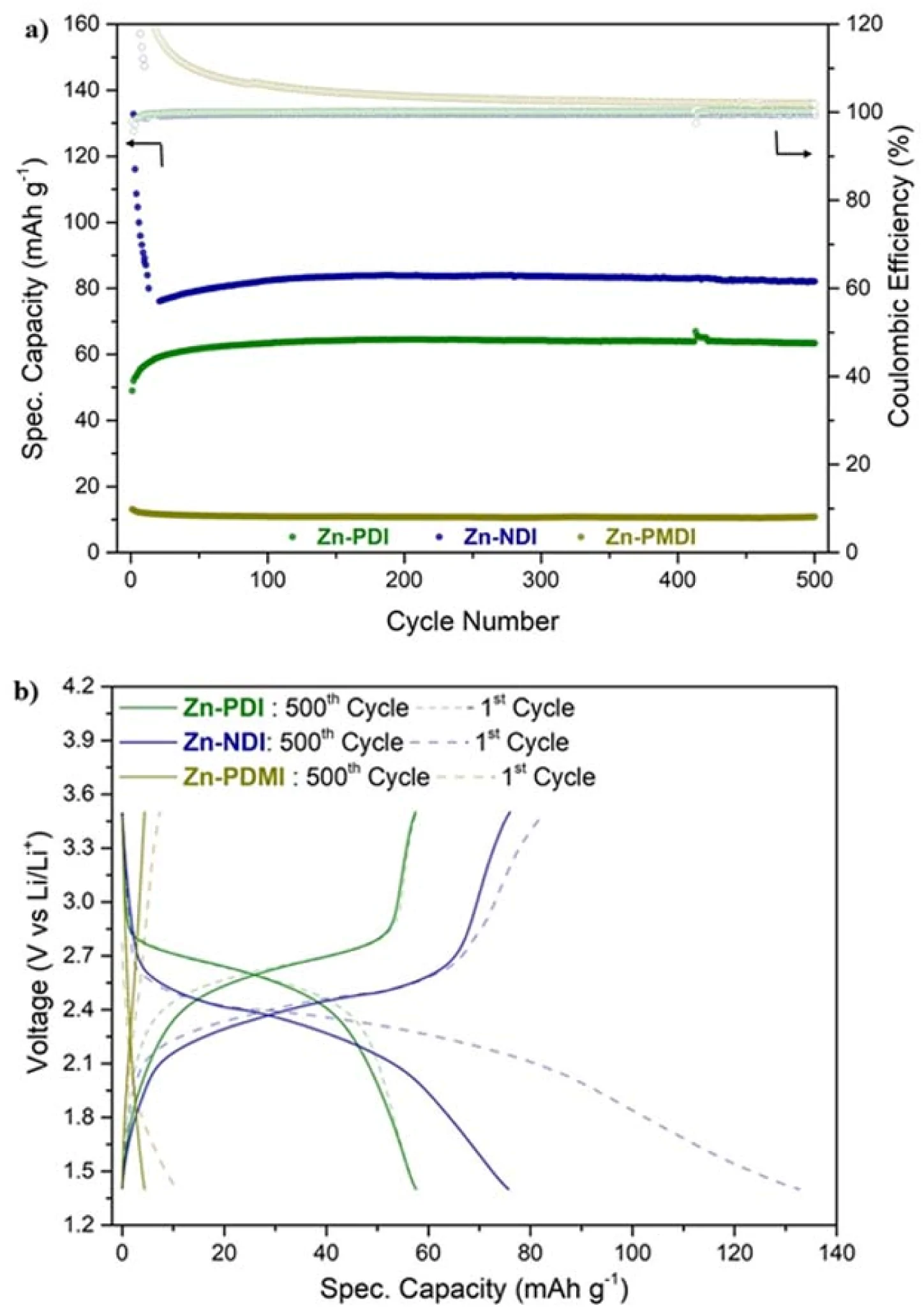
(a) Cycling stability and coulombic efficiency and (b) selected galvanostatic profiles of Zn-PDI, Zn-NDI, and Zn-PMDI using 1 M LiTFSI in DOL : DME (1 : 1) as electrolyte, at a C-rate of 2C within a potential window of 1.4–3.5 V *vs.* Li/Li^+^ for 500 cycles.

Cyclic voltammetry (CV) of Zn-PDI and Zn-NDI was measured at a scan rate of 1.0 mV s^−1^ over a potential range of 1.4–3.5 V *vs.* Li/Li^+^ (Fig. 4a). The CV profiles of both materials exhibit a single redox peak, corresponding to the successive reduction of the PDI and NDI moieties to the radical anion and dianion species. These peaks are centered at 2.6 V and 2.5 V *vs.* Li/Li^+^ for Zn-PDI and Zn-NDI, respectively. The CV results indicate that the charge storage mechanism of both redox-active MOFs is dominated by a faradaic process. After 1000 cycles at a rate of 2C, the discharge capacities reached 64 and 82 mAh g^−1^ for Zn-PDI and Zn-NDI, respectively (Fig. 4b), demonstrating excellent long-term cycling stability. Although some NDI-based molecular materials exhibit higher specific capacities, the electrochemical performance of Zn-NDI and Zn-PDI is within the range reported for several NDI- and PDI-based polymeric materials (Table S1).

**Fig. 4:**
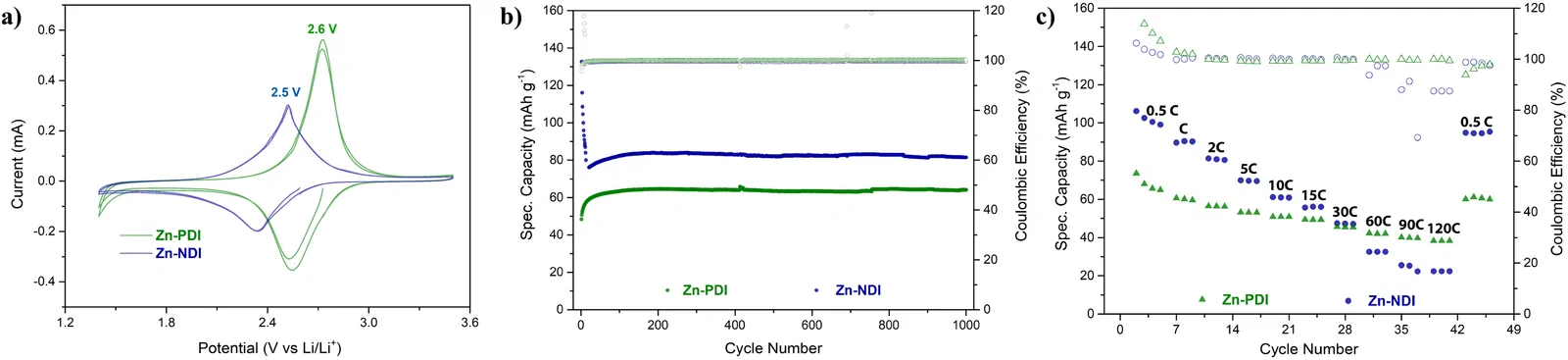
(a) Cyclic voltammetry of Zn-NDI and Zn-PDI MOFs measured in 1 M LiTFSI in DOL/DME (1 : 1, v/v) at a scan rate of 1.0 mV s^−1^. (b) Cycling stability and coulombic efficiency of Zn-NDI and Zn-PDI using 1 M LiTFSI in DOL/DME (1 : 1, v/v) as the electrolyte, evaluated at a C-rate of 2C within a potential window of 1.4–3.5 V *vs.* Li/Li^+^ over 1000 cycles. (c) Rate capability of Zn-NDI and Zn-PDI as a function of cycle number within a potential window of 1.4–3.5 V, at C-rates ranging from 0.5C to 120C.

Rate capability measurements were conducted to evaluate the electrochemical performance of the Zn-NDI and Zn-PDI electrodes at varying current densities. The tests were performed within a potential window of 1.4–3.5 V using current rates (C-rates) ranging from 0.5C to 120C, as shown in Fig. 4c. Zn-PDI exhibited higher discharge capacities at elevated rates (60C, 90C, and 120C), consistent with the kinetic analysis (see below), which indicated a predominantly capacitive charge storage contribution for this material. The more pronounced capacity loss of Zn-NDI at high rates is associated with its greater diffusion-controlled contribution (see below), whereas the higher capacitive contribution of Zn-PDI facilitates faster charge storage and superior rate capability. Notably, both materials recovered their initial capacities when the current rate was returned to 0.5C, demonstrating excellent electrochemical stability and reversibility.

### Charge storage mechanism

The lithiation mechanism was further investigated by CV at various scan rates ranging from 0.1 to 1.0 mV s^−1^ for both Zn-NDI and Zn-PDI (Fig. 5a and b). The power-law relationship (*i* = *aν*^*b*^) provides mechanistic insight, where the exponent *b* reflects the dominant charge-storage process: *b* values close to 0.5 indicate diffusion-controlled behavior, whereas *b* values approaching 1.0 suggest a surface-controlled (pseudocapacitive) mechanism.^45^ Analysis of the logarithmic plots of peak current *versus* scan rate (Fig. S14) shows that Zn-NDI exhibits comparatively lower *b* values (0.85 and 0.93 for the cathodic and anodic peaks, respectively), indicating a greater contribution from diffusion-limited processes. In contrast, Zn-PDI displays *b* values close to unity (0.96 and 1.00 for the cathodic and anodic peaks, respectively), highlighting a predominantly capacitive charge storage contribution.

**Fig. 5:**
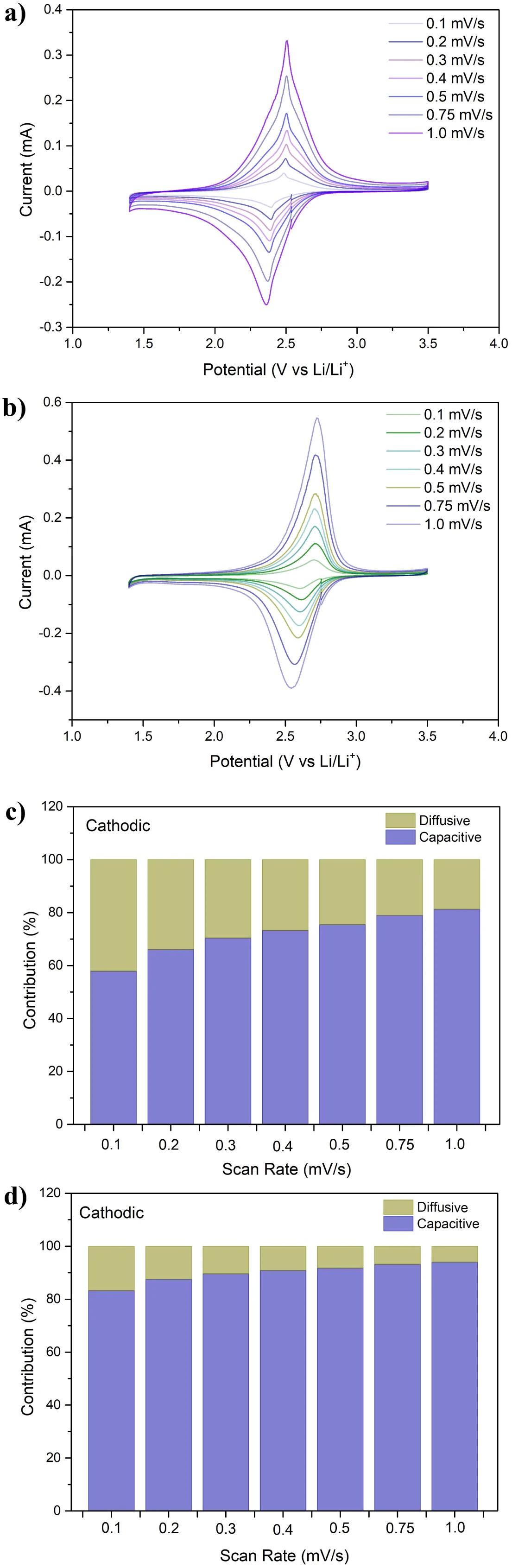
Scan rate-dependent CV at room temperature recorded at various scan rates ranging from 0.1 mV s^−1^ to 1 mV s^−1^ of (a) Zn-NDI and (b) Zn-PDI. Calculated capacitive-diffusive contributions (%) for the cathodic peak of (c) Zn-NDI and (d) Zn-PDI.

A quantitative analysis based on Dunn's method was performed to distinguish between capacitive and diffusive contributions at each redox peak (cathodic and anodic) for both Zn-NDI and Zn-PDI MOFs over a range of scan rates. This approach evaluates the current response at fixed potentials, where linear fitting is applied to determine the parameters *k*_1_ and *k*_2_ (Table S2). These parameters were subsequently used to quantify the respective capacitive and diffusion-controlled contributions (Fig. 5c, d and S15).

As expected, the capacitive contribution increases with increasing scan rate for both materials, owing to the higher current densities and shorter timescales, which limit lithium-ion diffusion. For the cathodic peak of Zn-NDI, the capacitive contribution increases from 58% at 0.1 mV s^−1^ to 81% at 1.0 mV s^−1^ (Fig. 5c). Notably, Zn-PDI consistently exhibits a higher capacitive contribution across all redox peaks, in agreement with its predominantly pseudocapacitive charge storage behavior and superior specific capacity at high rates (Fig. 4c).

The diffusion coefficients (*D*_Li_^^+^^) of Zn-NDI and Zn-PDI were determined using the Randles–Ševčík equation (eqn (S5)) for each redox peak over the investigated range of scan rates. In both materials, the diffusion coefficient associated with the anodic process (*D*_anodic_) is higher than that of the cathodic process (*D*_cathodic_) (Table S3), indicating faster lithium extraction compared to lithium insertion. For Zn-PDI, the calculated *D*_Li_^^+^^ values are slightly higher than those of Zn-NDI, indicating enhanced Li^+^ diffusion within the porous framework. This behavior can be attributed to the larger pore size of Zn-PDI, which likely facilitates more efficient Li^+^ diffusion through the channels.

### Post-mortem analysis

*Ex situ* X-ray photoelectron spectroscopy (XPS) was performed on pristine (before cycling) and cycled (after 1000 cycles) cathodes of Zn-NDI and Zn-PDI (Fig. 6 and 7) to further investigate the chemical and structural stability of the electrodes. For the pristine Zn-NDI electrode (Fig. S16b), the C 1s spectrum displays four principal components at 284.8, 285.9, 289.1, and 290.9 eV, corresponding to C–C, C–N/C–O, C=O, and O–C=O environments, respectively. An additional signal at 293.0 eV is assigned to C–F species arising from the PVDF binder. The C 1s spectrum is consistent with the expected molecular structure of the electrode prior to lithiation. The Zn 2p spectrum of the pristine electrode (Fig. 6a) exhibits two characteristic peaks at 1022.9 eV (Zn 2p_3/2_) and 1045.9 eV (Zn 2p_1/2_). The spin–orbit splitting of approximately 23.0 eV is consistent with Zn^2+^. Furthermore, the N 1s spectrum (Fig. S16a) shows two distinct components at 399.9 and 401.3 eV, which can be attributed to chemically differentiated nitrogen atoms within the pyrazole moiety upon coordination to Zn^2+^. These features support successful metal coordination to the NDI linker.

**Fig. 6:**
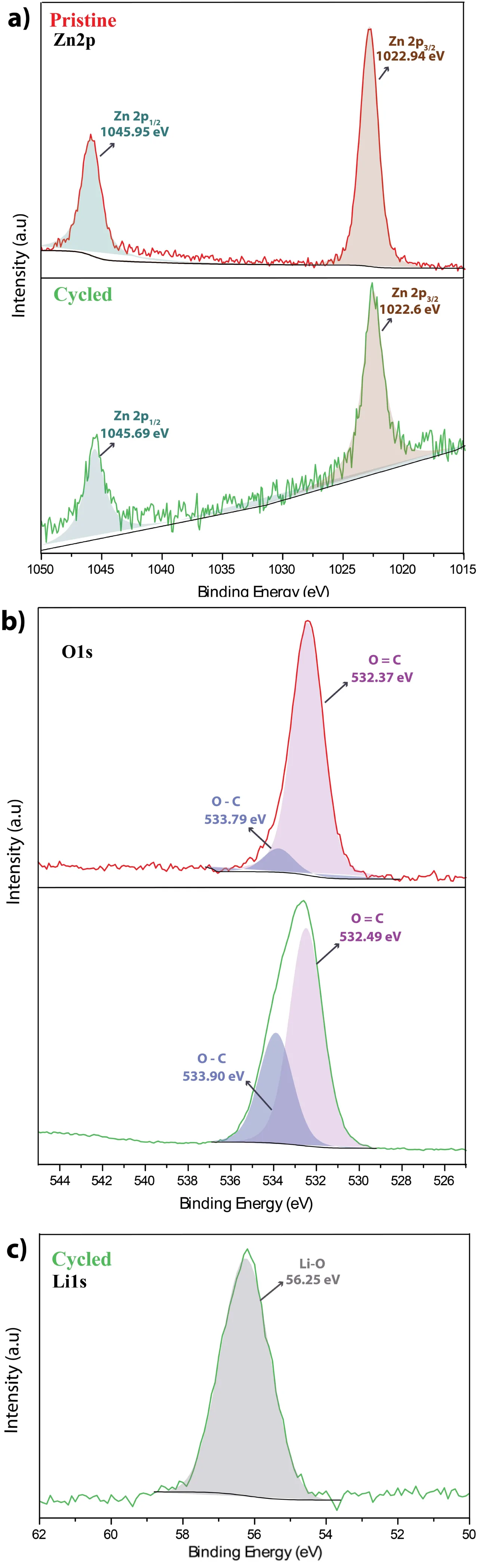
*Ex situ* XPS of Zn-NDI MOF (pristine and after 1000 cycles) electrodes: (a) Zn 2p, (b) O 1s, and (c) Li 1s spectra.

**Fig. 7:**
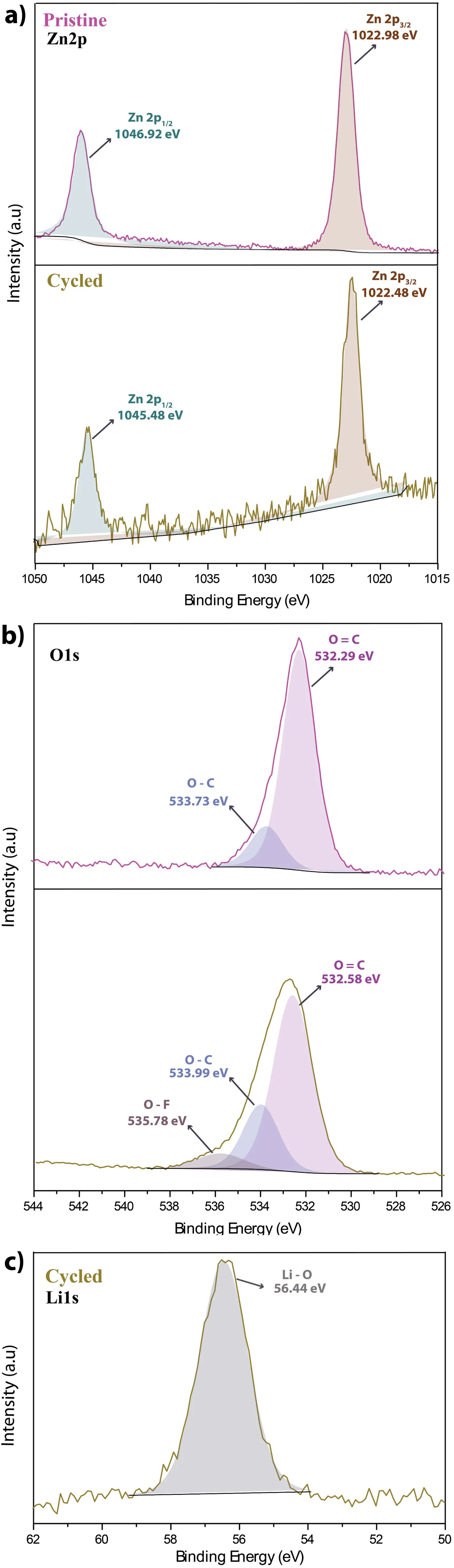
*Ex situ* XPS of Zn-PDI MOF (pristine and after 1000 cycles) electrodes: (a) Zn 2p, (b) O 1s, and (c) Li 1s spectra.

Upon full cycling of Zn-NDI (Fig. S16b), the C 1s spectrum shows a general decrease in peak intensity while largely retaining the same spectral profile as the pristine electrode, indicating preservation of the organic backbone. In the O 1s region (Fig. 6b), the peak at 532.5 eV, assigned to C=O, decreases in intensity, whereas the component at 533.9 eV, attributed to C–O species, increases. This change is consistent with a modification of the oxygen chemical environment upon lithiation/delithiation and may be associated with Li^+^ coordination to the oxygen-containing groups. However, contributions from cycling-induced surface/interphase species cannot be excluded. Importantly, the Zn 2p spectrum shows no significant changes in the positions of the Zn 2p_3/2_ and Zn 2p_1/2_ peaks, and the spin–orbit splitting remains essentially constant (Δ*E* ≈ 23.1 eV), confirming the persistence of Zn^2+^ after cycling. The absence of a significant shift in the Zn 2p signal provides no evidence for partial reduction of Zn^2+^ centres under the investigated conditions. These results, together with the electrochemical and theoretical analyses, support the assignment of the reversible redox processes primarily to the NDI linker rather than to the Zn nodes.

The pristine Zn-PDI electrode (Fig. 7) exhibits similar behavior to that observed for Zn-NDI. However, Zn-PDI contains additional chlorine atoms. In the pristine material (Fig. S17c), the Cl 2p spectrum displays two peaks at 201.1 and 202.7 eV, corresponding to Cl 2p_3/2_ and Cl 2p_1/2_, respectively. The Zn 2p spectrum (Fig. 7a) shows peaks at 1022.9 and 1045.9 eV, assigned to Zn 2p_3/2_ and Zn 2p_1/2_, with a spin–orbit splitting of approximately 23.0 eV, consistent with Zn^2+^. After cycling, the C 1s spectrum of Zn-PDI (Fig. S17b) similarly shows a decrease in overall intensity while maintaining its general profile, indicating preservation of the organic backbone. In the O 1s region (Fig. 7b), an additional component appears at 535.78 eV, which can be attributed to S=O/sulfonyl or other decomposition species, likely originating from the LiTFSI electrolyte. The appearance of this component, together with the changes observed in the O 1s region, indicates that cycling induces modifications to the surface chemical environment, potentially associated with the formation of electrolyte-derived interphase species. In the Cl 2p spectrum (Fig. S17c) after cycling, some additional components are observed, suggesting possible changes in the chemical environment of the chlorine species upon cycling. As with Zn-NDI, the Zn 2p spectrum of cycled Zn-PDI (Fig. 7a) shows no significant shift in Zn 2p_3/2_ and Zn 2p_1/2_ peak positions, and the spin–orbit splitting remains essentially unchanged (Δ*E* ≈ 23.0 eV), further supporting the preservation of Zn^2+^. Overall, the post-mortem XPS results indicate that, despite changes in the surface chemical environment upon cycling, the oxidation state of the Zn nodes and the main chemical features of the organic framework are largely preserved.

To further elucidate structural changes upon lithiation, *ex situ* PXRD was performed on Zn-NDI and Zn-PDI MOF electrodes in both pristine and cycled states (Fig. S18a and b). The *ex situ* PXRD pattern of the pristine Zn-NDI electrode (Fig. S18a) closely matches that of the electrode after 1000 cycles, preserving most of its characteristic diffraction peaks and demonstrating excellent structural integrity and stability throughout the lithiation/delithiation process. Similarly, Fig. S18b presents the *ex situ* PXRD patterns of the Zn-PDI electrodes. The pristine electrode exhibits a diffraction pattern comparable to that of the cycled one, with the characteristic reflections largely retained. Although the first diffraction peak is absent in both pristine and cycled samples, the remaining low-angle reflections are clearly preserved, suggesting that the crystalline framework of Zn-PDI was preserved after repeated charge–discharge cycles. The structural and morphological stability of the MOFs upon cycling was further evaluated by *ex situ* Raman spectroscopy and SEM (Fig. S19–S21). After 500 cycles, the characteristic vibrational features and morphology were largely preserved, providing evidence for the structural robustness of both Zn-NDI and Zn-PDI during prolonged electrochemical cycling.

### Quantum-chemical calculations

Theoretical calculations based on density functional theory (DFT) were performed for the Zn-XDI family of MOFs and the constituting ligands. Minimum-energy geometries of the isolated linkers calculated at the HSE06/6-31G(d,p) level of theory indicate that the arylene-diimide core unit tends to adopt a flat structure for PMDI and NDI, while PDI is twisted *ca.* 38° along the longitudinal axis due to the steric and electronic repulsion between proximal chlorine substituents (Fig. S22). In the three ligands, the pyrazole groups rotate along the C–N imide bond *ca.* 60° to reduce the steric congestion between the methyl-substituted pyrazole and the diimide.

The highest-occupied molecular orbital (HOMO) is calculated to be very similar in energy for all the ligands (−6.28, −6.26, and −6.24 eV for PMDI, NDI, and PDI, respectively). Whereas PMDI and NDI present a pyrazole-centered HOMO, the extended π-conjugation of PDI pushes its core-centered orbital slightly higher in energy than that of pyrazole (Fig. 8a). The lowest-unoccupied molecular orbital (LUMO) is calculated over the aromatic core in the three ligands, and its energy drops as the π-conjugated system size increases (from −3.45 eV in PMDI to −3.69 eV in NDI, and to −4.06 eV in PDI; Fig. 8a), in good accord with the voltammetry experiments. The LUMO stabilization predicted for PDI compared to NDI stems from the presence of the electron-withdrawing Cl substituents despite the π-core twisting (Table S4), as well as the larger π-system of PDI.^32^ First and second electron affinities at the same level of theory in solvent conditions are calculated to be −3.06, −3.29, and −3.70 eV, and −1.61, −2.08, and −2.86 eV for PMDI, NDI, and PDI, respectively. These results confirm that reduction is facilitated by extending the π-conjugation, making PDI a promising linker for MOF-based cathode materials.

**Fig. 8:**
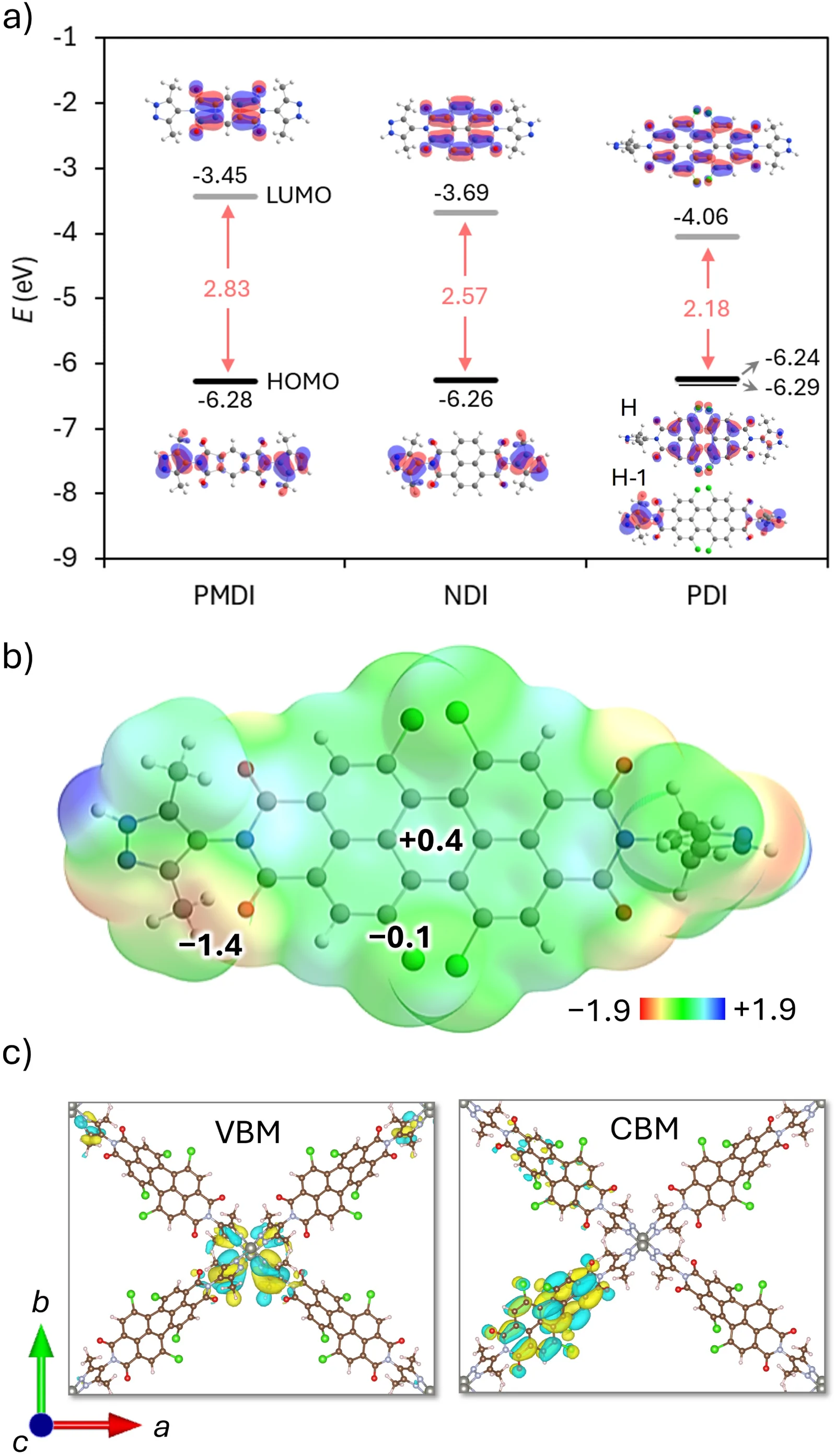
(a) Frontier molecular orbital energy diagram as calculated at the HSE06/6-31G(d,p) level of theory. The topology of the orbitals (isovalue of 0.03 a.u.) and eigenvalues are displayed. H and H−1 stand for HOMO and HOMO−1, respectively. (b) Molecular electrostatic potential map calculated for PDI. The color scale and relevant energy values of the surface are given in eV. (c) Frontier crystal orbitals corresponding to the valence band maximum (VBM) and conduction band minimum (CBM) for Zn-PDI MOF.

Molecular electrostatic potential maps feature clear potential wells at diimide oxygen atom positions (Fig. 8b for PDI, and Fig. S23 for PMDI and NDI), supporting that the interaction of the MOFs with Li^+^ proceeds preferentially through the lone pairs of the carbonyl oxygen, as indicated by the *ex situ* XPS experiments (Fig. 7c). Simulation of Li near the carbonyl oxygen leads to spontaneous reduction of XDI and Li^+^ formation, as evidenced by the charge analysis (Table S5) and the spin density (Fig. S24). The most negative Li–ligand binding energy is predicted for PDI (Table S6), confirming the increased stability experienced by the extra electron in the π-extended aromatic core. Theoretical calculations indicate that interaction of the ligands with a second Li^+^ ion will occur preferentially in a diagonal configuration, forming a doubly charged singlet electronic state XDI^2−^ (Table S7).

Finally, periodic boundary conditions calculations were performed for Zn-XDI MOFs to confirm the trends predicted on their constituting ligands. Minimum-energy crystal structures were obtained at the PBEsol level of theory (Table S8), mirroring the structural features predicted in the isolated ligands. DFT calculations at the HSE06 level indicate that the valence band maximum (VBM) is centered at the pyrazoles, and the conduction band minimum (CBM) is delocalized over the aromatic core of the ligand (Fig. 8c for Zn-PDI and Fig. S25 for Zn-PMDI and Zn-NDI), in agreement with the results obtained at the molecular level, with virtually no participation of the Zn species. The electronic structure in the Zn-XDI family is characterized by flat bands near the bandgap (Fig. S26), with only a moderate dispersion along the *Γ*–*Z* direction, which results from partial delocalization of the pyrazole-centered orbitals assisted by the –(Zn–N–N–)_∞_ chain. These results are consistent with the localized faradaic redox process at the diimide units upon Li^+^ insertion, as experimentally observed for the Zn-XDI series. The Hartree potential of Zn-XDI MOFs further supports the imide O as the primary site for Li^+^ interaction in the MOFs during the charge–discharge process (Fig. S27). Aligning the band structure with respect to the vacuum level^46^ leads to CBM values going from −3.92 eV in Zn-PMDI to −4.15 eV in Zn-NDI and to −4.39 eV in Zn-PDI. This trend confirms that the reducibility of Zn-XDI increases as the π-conjugation of the organic linker is extended.

## Conclusions

In summary, we have systematically investigated the influence of arylene diimide aromaticity on the electrochemical performance of a family of isoreticular Zn-XDI MOFs as cathode materials for lithium-ion storage. By varying the aromatic core from PMDI to NDI and PDI while maintaining the same framework topology, new structure–performance relationships in terms of specific capacity and cycling stability have been established. Among them, Zn-PDI and Zn-NDI exhibited a capacity retention of 98% and 87% after 500 cycles at 2C, whereas Zn-PMDI suffered from poor capacity retention, highlighting the critical role of the aromatic core in stabilizing reduced states during repeated lithiation and delithiation. Kinetic analyses demonstrated that Zn-PDI benefits from a greater capacitive contribution and improved Li^+^ transport, consistent with its improved rate capability performance. Post-mortem XPS and PXRD analyses confirmed that both Zn-NDI and Zn-PDI maintain their structural integrity. Complementary DFT calculations showed that increasing π-conjugation lowers the LUMO energy, increases electron affinity, and stabilizes lithiated intermediates, thereby facilitating reversible charge storage. Future studies could explore redox-active metal nodes as an additional design parameter, potentially enhancing both charge-storage capacity and electronic conductivity in redox-active MOFs. Overall, this work provides valuable guidelines for the development of next-generation organic and MOF-based electrode materials based on arylene diimide moieties with enhanced cycling stability, specific capacity, and rate performance.

## Experimental

### General methods and materials

^1^H NMR spectra were recorded on a JEOL 400 MHz spectrometer at 293 K. Proton chemical shifts (*δ*, ppm) were referenced to the residual non-deuterated solvent signal as an internal standard (DMSO-*d*_6_: *δ* = 2.50 ppm). ^1^H NMR data are reported as follows: *δ* (ppm) (multiplicity, integration).

Powder X-ray diffraction (PXRD) analyses were performed on a Siemens D5000 Kristalloflex diffractometer equipped with a Göbel mirror (Bruker) using monochromatic Cu Kα radiation (*λ* = 1.5406 Å) operating at 45 kV and 40 mA. PXRD data were collected over a 2*θ* range of 3–30° with a step size of 0.017°.

The Brunauer–Emmett–Teller (BET) surface areas and pore size distributions of the Zn-XDI frameworks were determined using vacuum-dried powder samples. Nitrogen sorption isotherms were measured at 77 K on a Micromeritics ASAP 2060 instrument. Prior to measurement, the samples were activated at 140 °C under a nitrogen flow using a Micromeritics FlowPrep 060 sample preparation unit.

Thermogravimetric analysis (TGA) of the powder samples was conducted employing a TA Instruments Q5000 IR thermobalance. The TGA measurements involved a general heating profile ranging from 25 to 800 °C, with a heating rate of 5 °C min^−1^ under an N_2_ atmosphere using a gas flow rate of 25 mL min^−1^.

Scanning electron microscopy (SEM) images were recorded using a FESEM equipment (ultra plus, Zeiss, Germany).

Raman spectra were recorded using a WITec alpha300R confocal Raman microscope.

#### Electrochemical cell assembly and analysis

The working electrodes were fabricated by first dry grinding the MOFs as active materials (50 wt%), carbon black (CB) as the conductive agent (40 wt%) and polyvinylidene fluoride (PVDF, powder, Sigma-Aldrich) as the binder (10 wt%) in a mortar. NMP was subsequently added to form a homogeneous slurry after thorough milling. After milling, the slurry was cast onto carbon-coated Al foil using a doctor blade. After drying under vacuum for 24 h at 80 °C, the electrodes were punched into 12 mm disks and dried overnight at 50 °C before being transferred into the glovebox. The mass loading of the electrodes was between 0.4–0.9 mg cm^−2^.

The electrochemical tests were performed in half-cells (CR2032, SS316, coin cell format) using metallic Li as both the counter and pseudo-reference electrode and a sheet of glass microfiber filters (Whatman, GF/D) as the separator. A 1 M LiTFSI solution in dioxolane and dimethoxyethane (1 : 1, v/v) was used as the electrolyte for Li cells. The assembly was carried out in an Ar-filled glovebox (MBraun, <0.1 ppm O_2_ and H_2_O). Galvanostatic charge–discharge tests were performed using a Neware battery testing system at ambient temperature. Cyclic voltammetry and electrochemical impedance spectroscopy were conducted using an impedance analyser (SP-150e Potentiostat, BioLogic).

#### Post-mortem studies

*Ex situ* XPS and PXRD analyses were performed on both pristine and cycled electrodes. The cycled samples were obtained by opening the coin cells inside a glovebox and rinsing them with ethylene carbonate (EC) and diethyl carbonate (DEC). The samples were then dried in a vacuum chamber and under an argon atmosphere. Both pristine and cycled electrodes were mounted in XPS and PXRD holders and analysed under argon. For XPS analysis, a Thermo Scientific Escalab 250 Xi spectrometer was used. For *ex situ* PXRD analysis, a Malvern Panalytical-Empyrean diffractometer was employed.

### Synthesis of pyrazole-XDI linkers (XDI = PDI, NDI, PMDI)

The pyrazole-XDI (XDI = PMDI, NDI, PDI) linkers were synthesized as described in the SI, and their purity was confirmed by ^1^H NMR spectroscopy. PMDI: ^1^H NMR (400 MHz, DMSO-*d*_6_) *δ* (ppm): 12.61 (s, 2H), 8.37 (s, 2H), 2.06 (s, 12H). NDI: ^1^H NMR (400 MHz, DMSO-*d*_6_) *δ* (ppm): 12.50 (s, 2H), 8.74 (s, 4H), 2.02 (s, 12H). PDI: ^1^H NMR (400 MHz, DMSO-*d*_6_) *δ* (ppm): 12.39 (s, 2H), 8.63 (s, 4H), 2.04 (s, 12H).

### Synthesis of Zn-XDI MOFs

The Zn-XDI frameworks were synthesized following a previously reported solvothermal procedure with minor modifications.^40^ In a typical preparation, 0.10 mmol of the corresponding pyrazole-XDI linker and 32.7 mg of Zn(NO_3_)_2_·6H_2_O (0.11 mmol) were added to 10 mL of DMF (in a 20 mL vial) and the mixture was sonicated to fully dissolve the reagents until a clear and homogeneous solution was obtained, then degassed with argon for 10 min. The vials were then sealed and placed in a gravity convection oven, heated slowly from room temperature to 135 °C (heating rate 5 °C min^−1^), and maintained at this temperature for 4.5 h. After that, the reaction mixture was allowed to cool slowly to room temperature. The obtained polycrystalline material (MOF) was isolated by centrifugation. The MOF powder was then soaked in fresh DMF for 1 h and centrifuged; this washing step was repeated two additional times. After the last centrifugation step, the subsequent solvent exchange was carried out by soaking the MOF powder in methanol for 3 h, followed by two additional exchanges with fresh methanol. The framework was isolated after each exchange by centrifugation. Finally, the MOF powder was dried under vacuum to afford the activated Zn-XDI framework.

### Computational details

First-principles calculations were performed using DFT. Geometry optimizations and frequency analyses of the isolated linkers were conducted in Gaussian-16(A.03)^47^ employing the hybrid HSE06 (ref. 48) functional combined with the 6-31G(d,p) basis set.^49^ Solvent conditions were implemented through the SMD continuum solvent model.^50^ Periodic bulk structures (atomic coordinates and lattice parameters) were relaxed in FHI-aims^51^ using the PBEsol functional^52^ and Tkatchenko–Scheffler van der Waals correction,^53^ using numerical atom-centered orbital basis sets. A hierarchical approach was adopted for these periodic relaxations, utilizing a “light” basis set for pre-optimization followed by refinement with an “intermediate” basis set. The Brillouin zone was sampled with *k*-point meshes of 3 × 3 × 6, 3 × 5 × 12, and 3 × 3 × 12 for Zn-PMDI, Zn-NDI, and Zn-PDI, respectively. Electronic densities of states were computed using the tetrahedron integration method, and scalar relativistic corrections were treated *via* the ZORA formalism (see the SI for full computational details).

## Author contributions

C. R. and J. C. performed all electrochemical characterization and post-mortem analysis of the materials. A. K. and S. O. synthesized and characterized the materials. A. N. G. and M. H. B. contributed to the analysis of electrochemical data. J. R. contributed to the discussion of the results. G. G.-P. performed the theoretical calculations and J. C. (UV) supervised the theoretical calculations section. M. S. conceived, designed and supervised the whole project. The manuscript was written through contributions of all authors. All authors have given approval to the final version of the manuscript.

## Conflicts of interest

There are no conflicts to declare.

## Supplementary Material

TA-014-D6TA05048D-s001

## Data Availability

Data for this article, including electrochemical data, XPS, PXRD, and computational data are available at Zenodo at https://doi.org/10.5281/zenodo.20666754. Supplementary information (SI): general methods and materials, synthesis and characterisation of Zn-XDI MOFs, electrochemical measurements, charge storage mechanism, post-mortem studies and computational details. See DOI: https://doi.org/10.1039/d6ta05048d.
