# Supplementary material for: Influence of arylene diimide linker aromaticity on the electrochemical performance of redox-active metal–organic framework cathodes for lithium-ion storage
Source: J Mater Chem A Mater. 2026 Oct 12. Online ahead of print. doi: 10.1039/d6ta05048d (PMC13657018; doi:10.1039/d6ta05048d)
Supplement: Supplementary file 1 [file TA-OLF-D6TA05048D-s001.pdf]

Supplementary Information (SI) for Journal of Materials Chemistry A.
This journal is © The Royal Society of Chemistry

Supporting Information

Influence of Arylene Diimide Linker Aromaticity on the Electrochemical Performance of Redox-Active Metal–Organic Framework Cathodes for Lithium-Ion Storage

Catarina Ribeiro,[a] Amol Kumar,[b] Jonathan Caroni,[c] Gioele García-Pinar,[d] A. Nuno Guerreiro,[e] Joao Rocha,[a] M. Helena Braga,[e,f] Joaquín Calbo,*[d] Sascha Ott,*[b] and Manuel Souto*[c,g,a]

[a] Department of Chemistry, CICECO-Aveiro Institute of Materials, University of Aveiro, Aveiro, 3810-393, Portugal
[b] Department of Chemistry - Ångström Laboratory, Uppsala University, 75120 Uppsala, Sweden
[c] CiQUS, Centro Singular de Investigación en Química Bioloxica e Materiais Moleculares, Departamento de Química-Física, Universidade de Santiago de Compostela, 15782, Santiago de Compostela, Spain
[d] Instituto de Ciencia Molecular (ICMol) Universidad de Valencia 46980 Paterna, Spain
[e] MatER, Faculty of Engineering, University of Porto, 4200-465 Porto, Portugal
[f] LAETA, Institute of Science and Innovation in Mechanical and Industrial Engineering
[g] Oportunius, Galician Innovation Agency (GAIN) 15702 Santiago de Compostela, Spain

Contents

1. **General methods and materials** S3
2. **Synthesis and characterization of Zn-XDI MOFs** S4
3. **Electrochemical measurements** S11
4. **Charge storage mechanism** S14
5. ***Post-mortem* studies** S17
6. **Computational details** S20
7. **References** S27

1. General methods and materials

All **reagents and solvents** employed in the syntheses were of high purity grade and were purchased from Sigma-Aldrich Co., or TCI.

^{1}H NMR measurements were performed on a JEOL 400 MHz spectrometer at 293 K. Proton chemical shifts are calibrated using residual non-deuterated solvent peaks as an internal reference (DMSO-d_6: 2.50). Data for ^{1}H NMR spectra are reported in the following way: chemical shift (δ in ppm) (multiplicity, integration).

Powder X-ray diffraction (PXRD) analysis was performed on a Siemens D5000 Kristalloflex equipped with a Gobel mirror (Bruker) using a monochromatic Cu Kα radiation (λ = 1.5406 Å) source operating at 45 kV and 40 mA. PXRD data was collected between 3 and 30º (2θ) range at a step size of 0.0170.

The **Brunauer-Emmett-Teller (BET)** surface areas and pore sizes of the Zn-XDI frameworks were determined on corresponding vacuum-dried powder sample. The measurements were carried out using a Micromeritics ASAP 2060 with N_2 sorption isotherm, recorded at 77 K. Before the measurement, the powder was activated at 140°C under a nitrogen flow using a Micromeritics FlowPrep 060 sample preparation unit.

Electrochemical cell assembly and analysis: The working electrodes were fabricated by dry grinding the MOFs as active materials (50 wt%), carbon black (CB) as the conductive agent (40 wt%) and polyvinylidene fluoride (PVDF, powder, Sigma–Aldrich) as the binder (10 wt%). The electrodes were prepared by mixing the active material, CB and PVDF in NMP. After milling, the slurry was cast onto carbon-coated Al foil using a doctor blade. After drying under vacuum for 24 h at 80 °C, the electrodes were pinched into 12 mm disks and dried overnight at 50 ºC before being transferred into the glovebox. The mass loading of the electrodes was between 0.4-0.9 mg cm^{-2}.

The electrochemical tests were performed in half-cells (CR2032, SS316, coin cell format) using metallic Li as both the counter and pseudo-reference electrode and a sheet of glass microfiber filters (Whatman, GF/D) as the separator. A 1M LiTFSI solution in dioxalane and dimethoxyethane (1:1, v/v) was used as the electrolytes for Li cells. The assembly was carried out in an Ar-filled glovebox (MBraun, <0.1 ppm O_2 and H_2O). Galvanostatic charge–discharge tests were performed using a Neware battery testing system at ambient temperature. Cyclic voltammetry and electrochemical impedance spectroscopy were conducted using an impedance analyser (SP-150e Potentiostat, BioLogic).

Post-mortem studies: *Ex-situ* XPS and PXRD analyses were performed on both pristine and cycled electrodes. The cycled samples were obtained by opening the coin cells inside a glovebox and rinsing them with ethylene carbonate (EC). The samples were then dried in a vacuum chamber and under an argon atmosphere. Both pristine and cycled electrodes were mounted in XPS and PXRD holders and analysed under argon. For XPS analysis, a Thermo Scientific Escalab 250 Xi spectrometer was used. For PXRD analysis, a Malvern Panalytical-Empyrean diffractometer was employed.

Thermogravimetric analysis (TGA): TGA of the powder samples was conducted employing a TA Instruments Q5000 IR thermobalance. The TGA measurements involved a general heating profile ranging from 25 to 800 °C, with a heating rate of 5 °C min^{-1} under an N_2 atmosphere using a gas flow rate of 25 mL min^{-1}.

SEM: Scanning electron microscopy (SEM) images were recorded using a FESEM equipment (ultra plus, Zeiss, Germany).

Raman spectroscopy: Raman spectra were recorded using a WITec alpha300R confocal Raman microscope.

2. Synthesis and characterization of Zn-XDI MOFs

Synthesis of pyrazole-XDI Linkers (X= P, N, PM)

The pyrazole-XDIs (X = PM, N, P) linkers were prepared following previously published protocols, and the purity of the linker was confirmed by ^{1}H NMR spectroscopy.

PMDI: ^{1}H NMR (400 MHz, DMSO-d_6) δ (ppm): 12.61 (s, 2H), 8.37 (s, 2H), 2.06 (s, 12H).

NDI: ^{1}H NMR (400 MHz, DMSO-d_6) δ (ppm): 12.50 (s, 2H), 8.74 (s, 4H), 2.02 (s, 12H).

PDI: ^{1}H NMR (400 MHz, DMSO-d_6) δ (ppm): 12.39 (s, 2H), 8.63 (s, 4H), 2.04 (s, 12H)

Synthesis of Zn-XDI MOFs

The **Zn-XDI** frameworks were synthesized following a previously reported solvothermal procedure with minor modifications.[1] In a typical preparation, 0.10 mmol of the corresponding pyrazole-XDI linker and 32.7 mg of $Zn(NO_3)_2{\cdot}6H_2O$ (0.11 mmol) were added to 10 ml of DMF (in a 20 mL vial) and the mixture was sonicated to fully dissolve the reagents until a clear and homogeneous solution was obtained, then degassed with argon for 10 min. The vials were then sealed and placed in a gravity convection oven, heated slowly from room temperature to 135 ºC (heating rate 5 ºC/min), and maintained at this temperature for 4.5 h. After that, the reaction mixture was allowed to cool slowly to room temperature.

The obtained polycrystalline material (MOF) was isolated by centrifugation. The MOF powder was then soaked in fresh DMF for 1h and centrifuged; this washing step was repeated two additional times. After the last centrifugation step, the subsequent solvent exchange was carried out by soaking the MOF powder in methanol for 3 h, followed by two additional exchanges with fresh methanol. The framework was isolated after each exchange by centrifugation. Finally, the MOF powder was dried under vacuum to afford the activated **Zn-XDI** framework. The crystallinity of the MOF was confirmed through PXRD analysis (see Figure 1).

The Brunauer-Emmett-Teller (BET) surface areas and pore sizes of the **Zn-XDI** frameworks were determined on vacuum-dried powder sample. As expected, the pore sizes increase from **Zn-PMDI** to Zn-NDI and **Zn-PDI** and the corresponding BET surface area follow the expected inverse trend.[2] In well-defined porous MOFs, an increase in pore diameter typically leads to decreases in overall surface area, assuming comparable framework topology and crystallinity.[3]

For the isoreticular **Zn-XDI** series, elongation of the organic linker expands the pore diameter but simultaneously reduces the number of pores per unit volume which in turn decrease the total accessible surface area.[4,5]

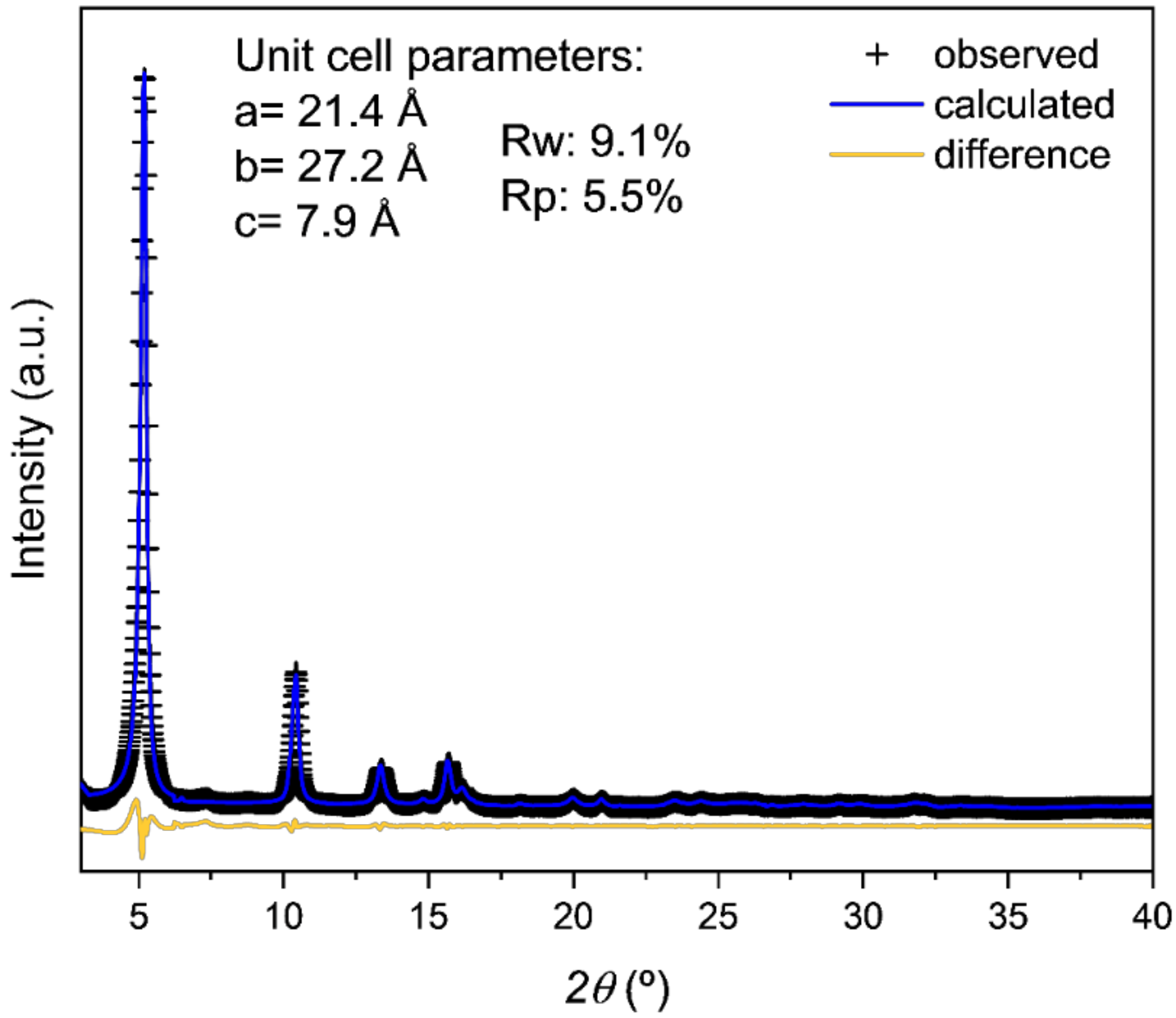

Figure S1. PXRD patterns and Pawley refinement of **Zn-PMDI**.

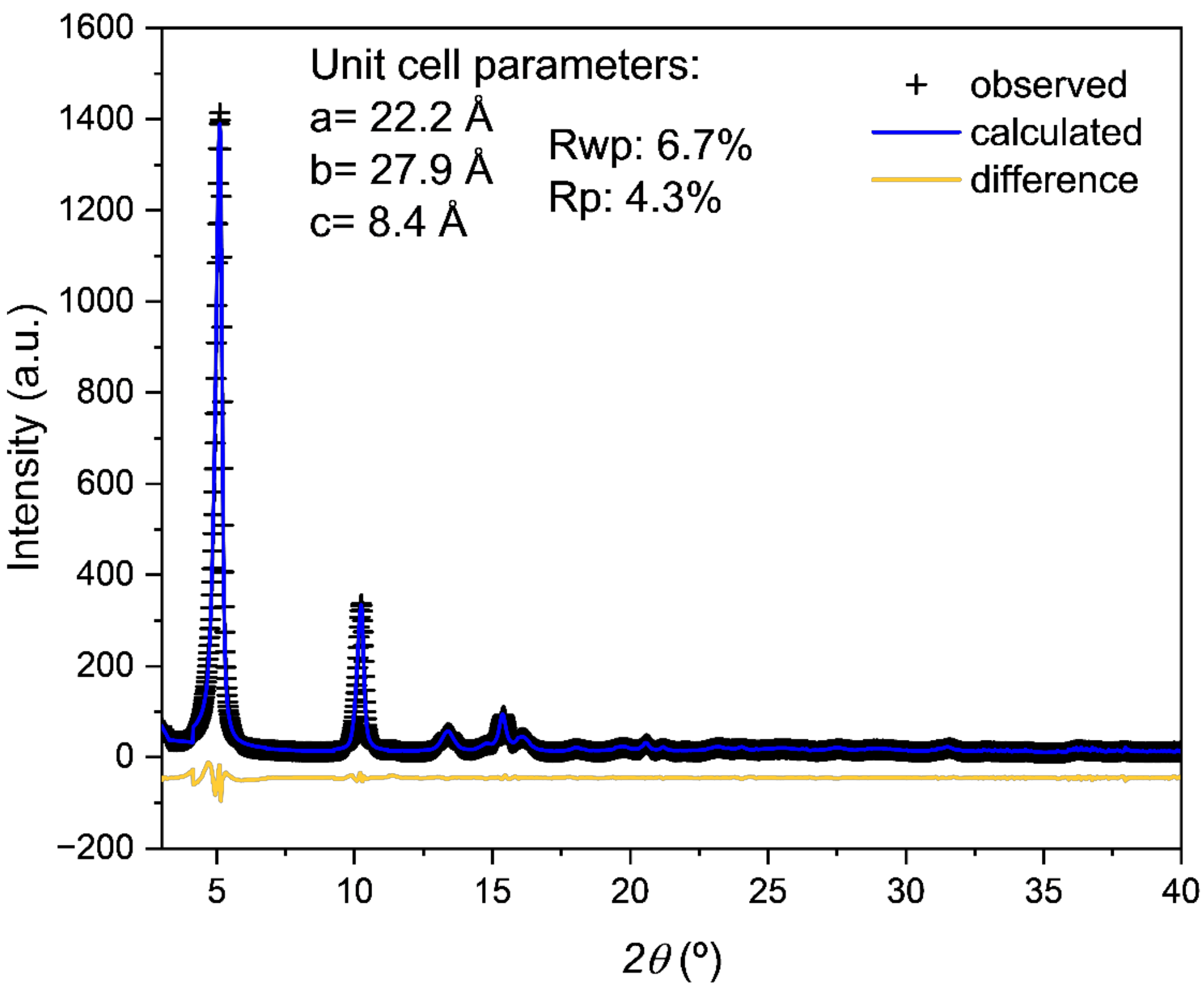

Figure S2. PXRD patterns and Pawley refinement of **Zn-NDI**.

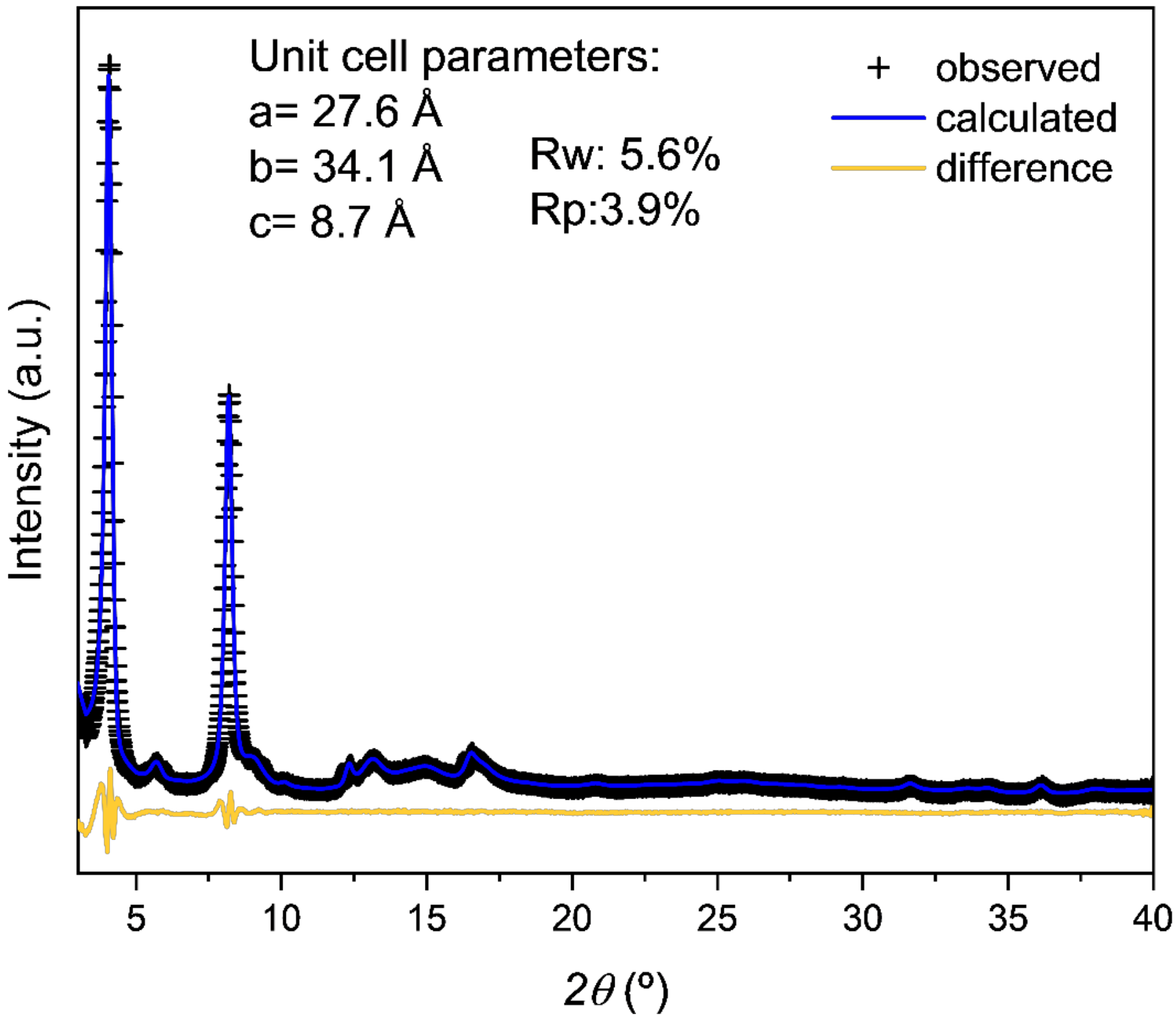

Figure S3. PXRD patterns and Pawley refinement of **Zn-PDI**.

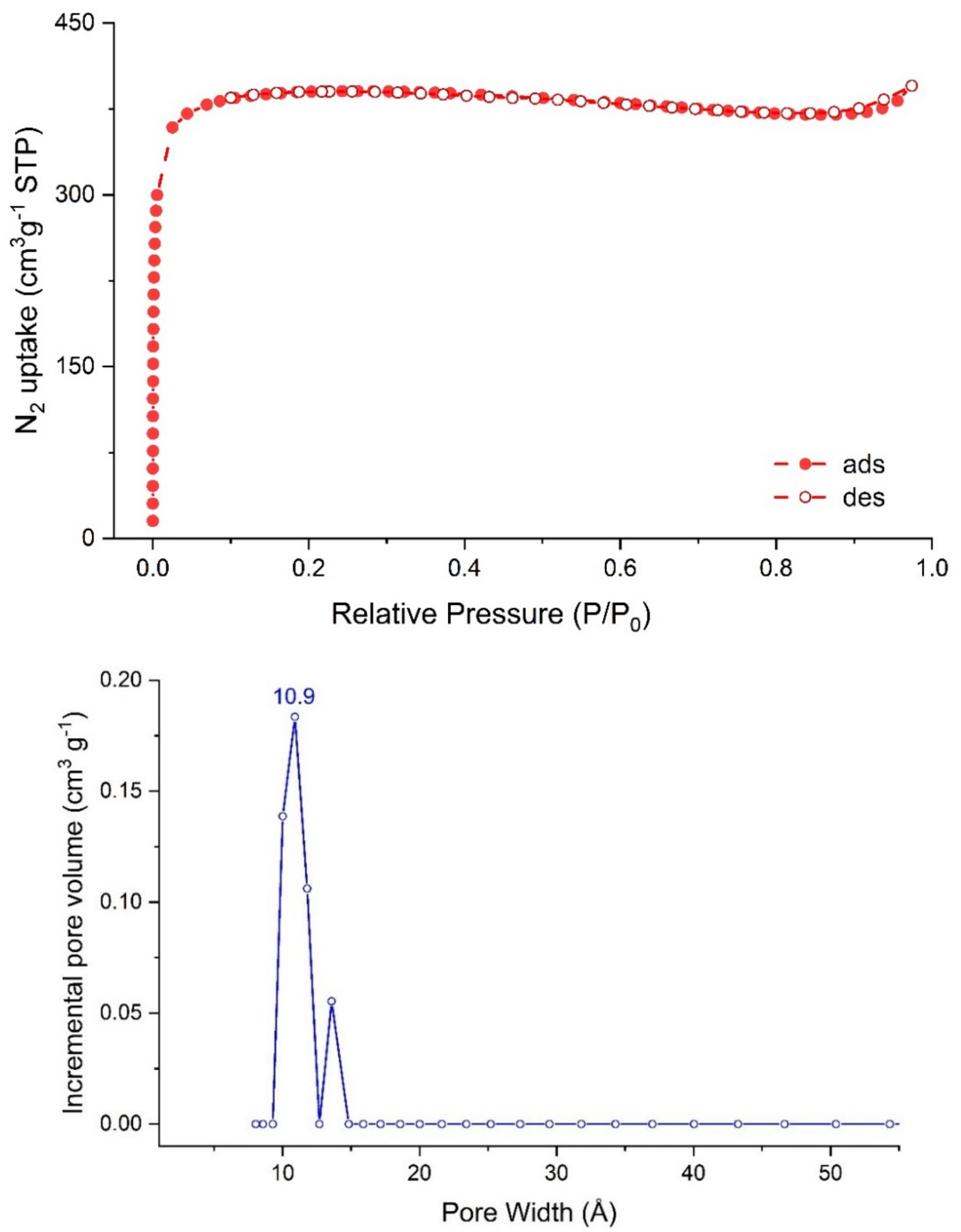

Figure S4. N_2 sorption isotherm at 77 K of **Zn-PMDI** (top) and corresponding pore width distribution (bottom) with BET surface area of 1180 ± 30 m²/g.

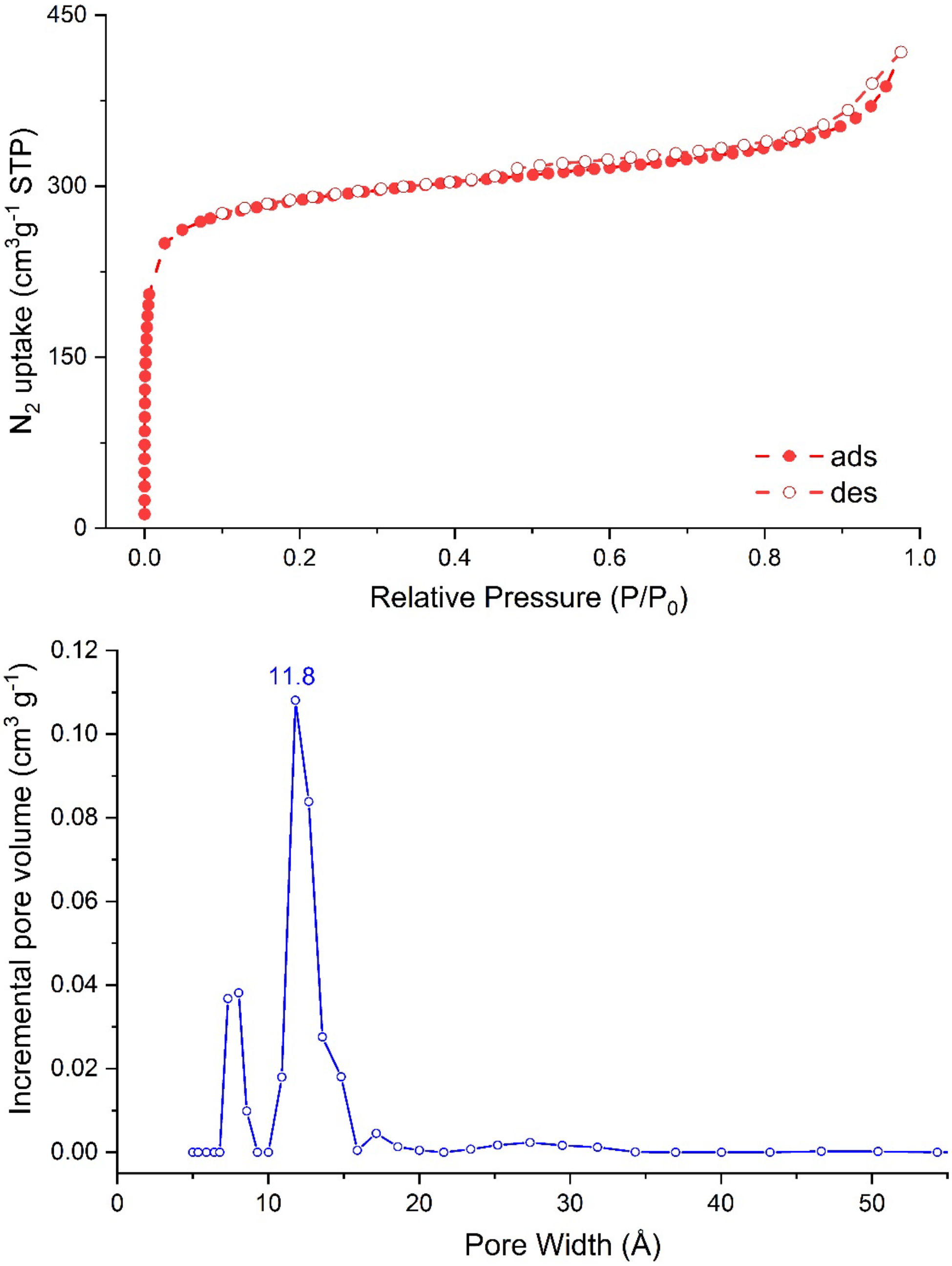

Figure S5. N_2 sorption isotherm at 77 K of **Zn-NDI** (top) and corresponding pore width distribution (bottom) with BET surface area of 900 ± 20 m²/g.

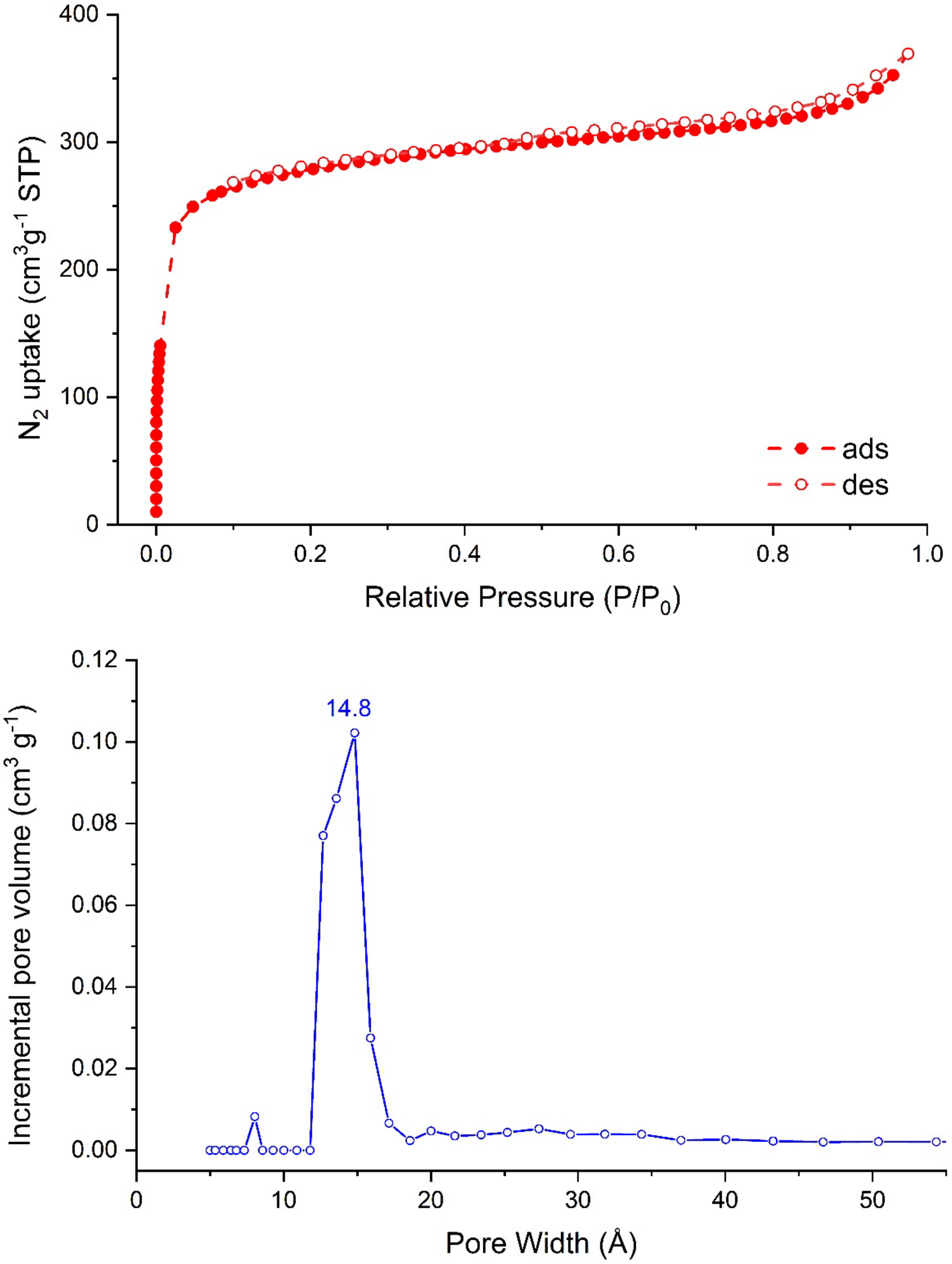

Figure S6. N_2 sorption isotherm at 77 K of **Zn-PDI** (top) and corresponding pore width distribution (bottom) with BET surface area of 875 ± 20 m²/g.

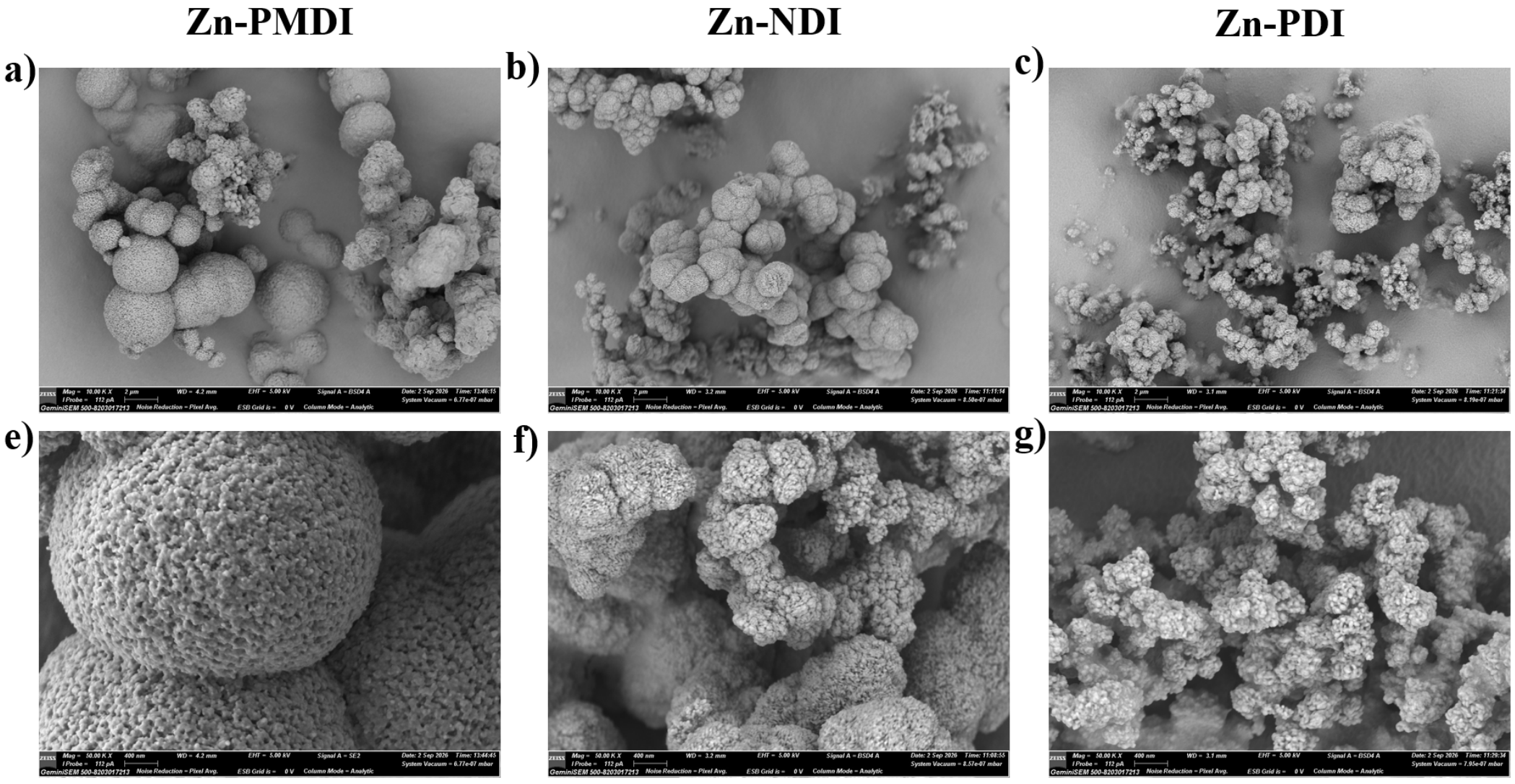

Figure S7. SEM images of **Zn-PMDI** (a and e), **Zn-NDI** (b and f), and **Zn-PDI** (c and g).

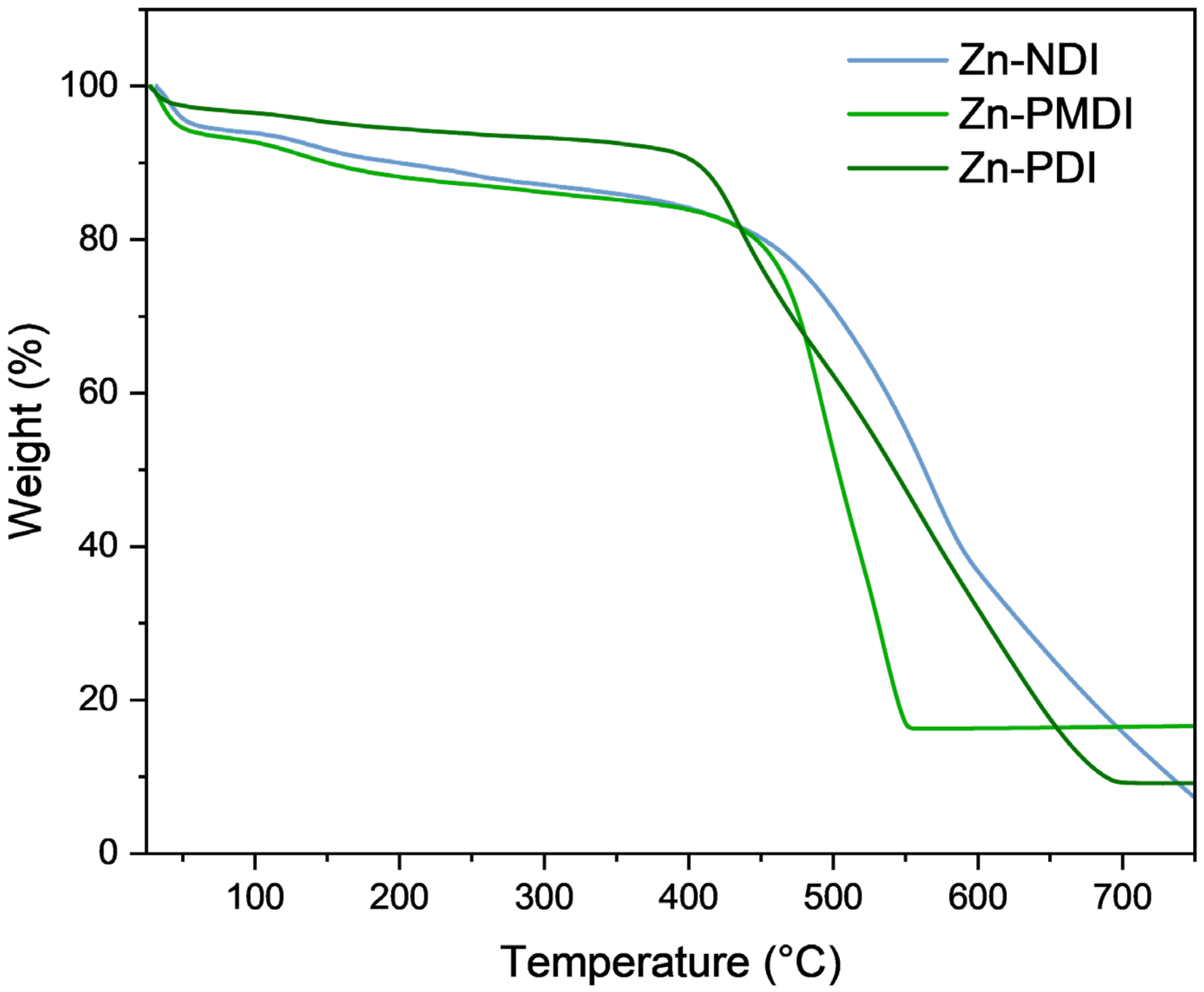

Figure S8. TGA profile of **Zn-PMDI**, **Zn-NDI**, and **Zn-PDI** at a heating rate of 5 °C min^{-1} under a constant stream of N_2.

3. Electrochemical measurements

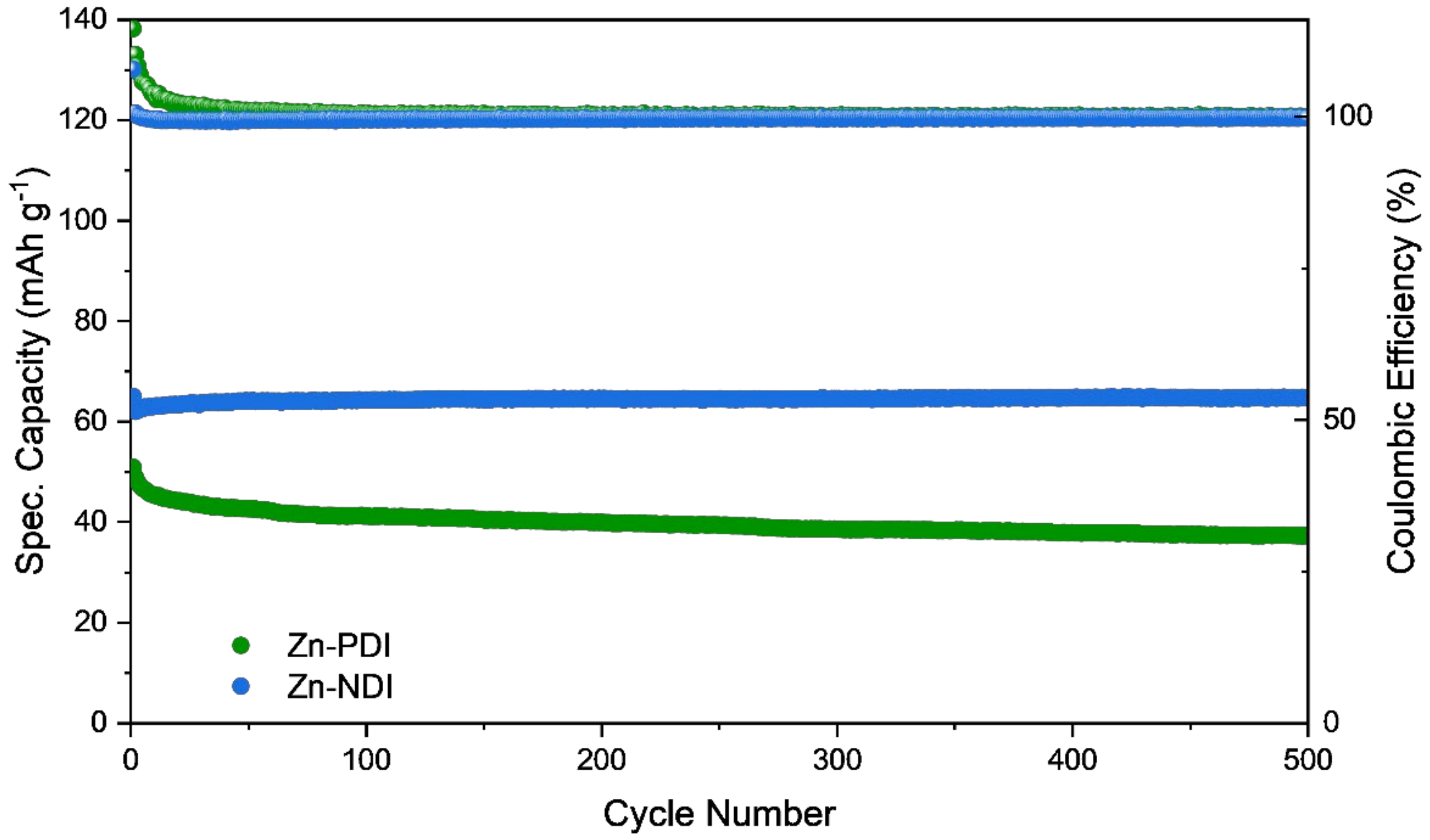

Figure S9. Cycling performance of **Zn–NDI** and **Zn–PDI** electrodes with a 60:30:10 wt% ratio of active material/conductive carbon/binder at 2C over 500 cycles.

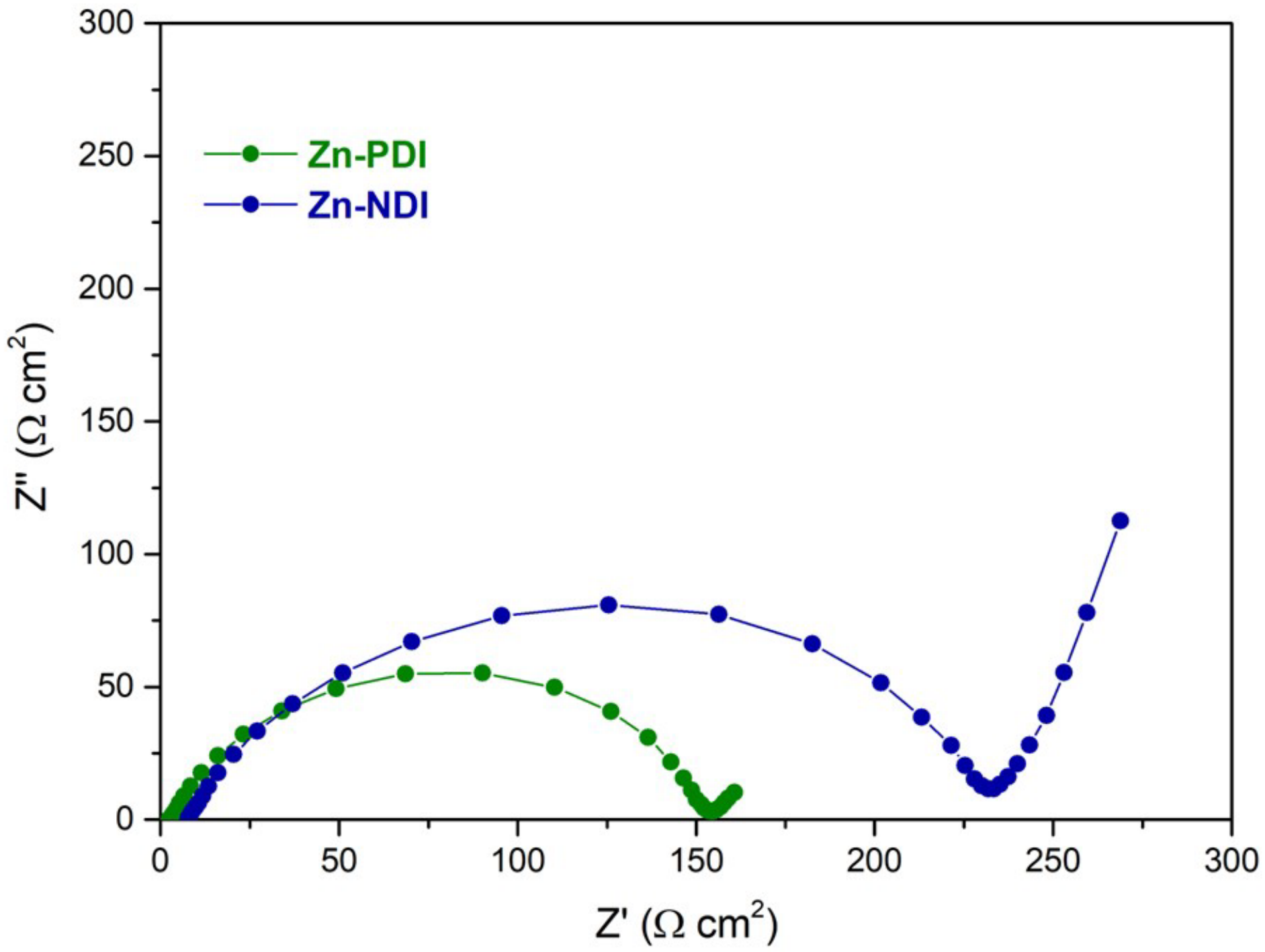

Figure S10. Potential electrochemical impedance spectroscopy (PEIS) of **Zn-NDI** and **Zn-PDI** MOFs, before charge.

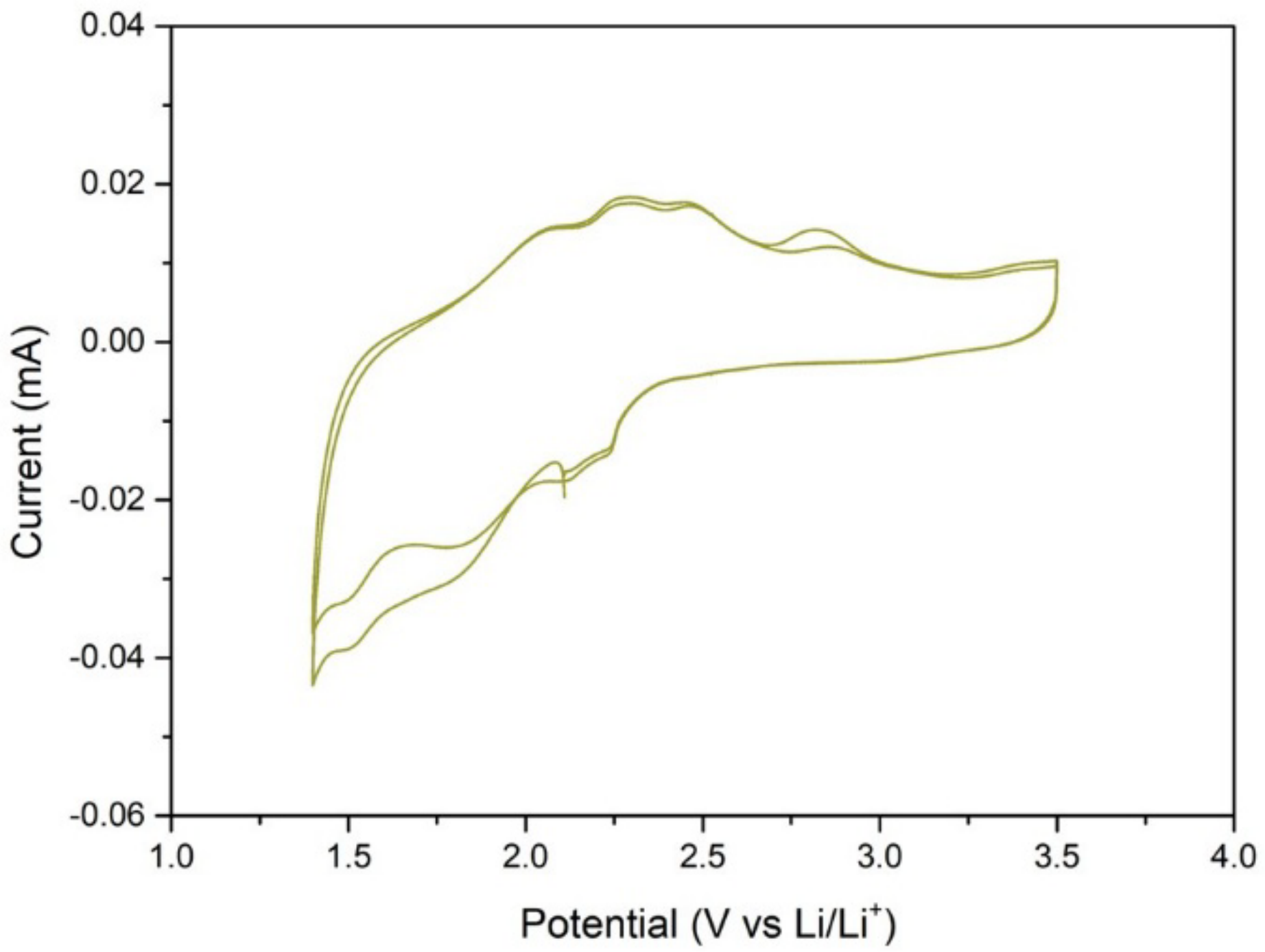

Figure S11. Cyclic voltammetry of **Zn-PMDI MOF** using 1 M LiTFSI in DOL/DME (1:1 vol) at a scan rate of 1.0 mV s^{-1}.

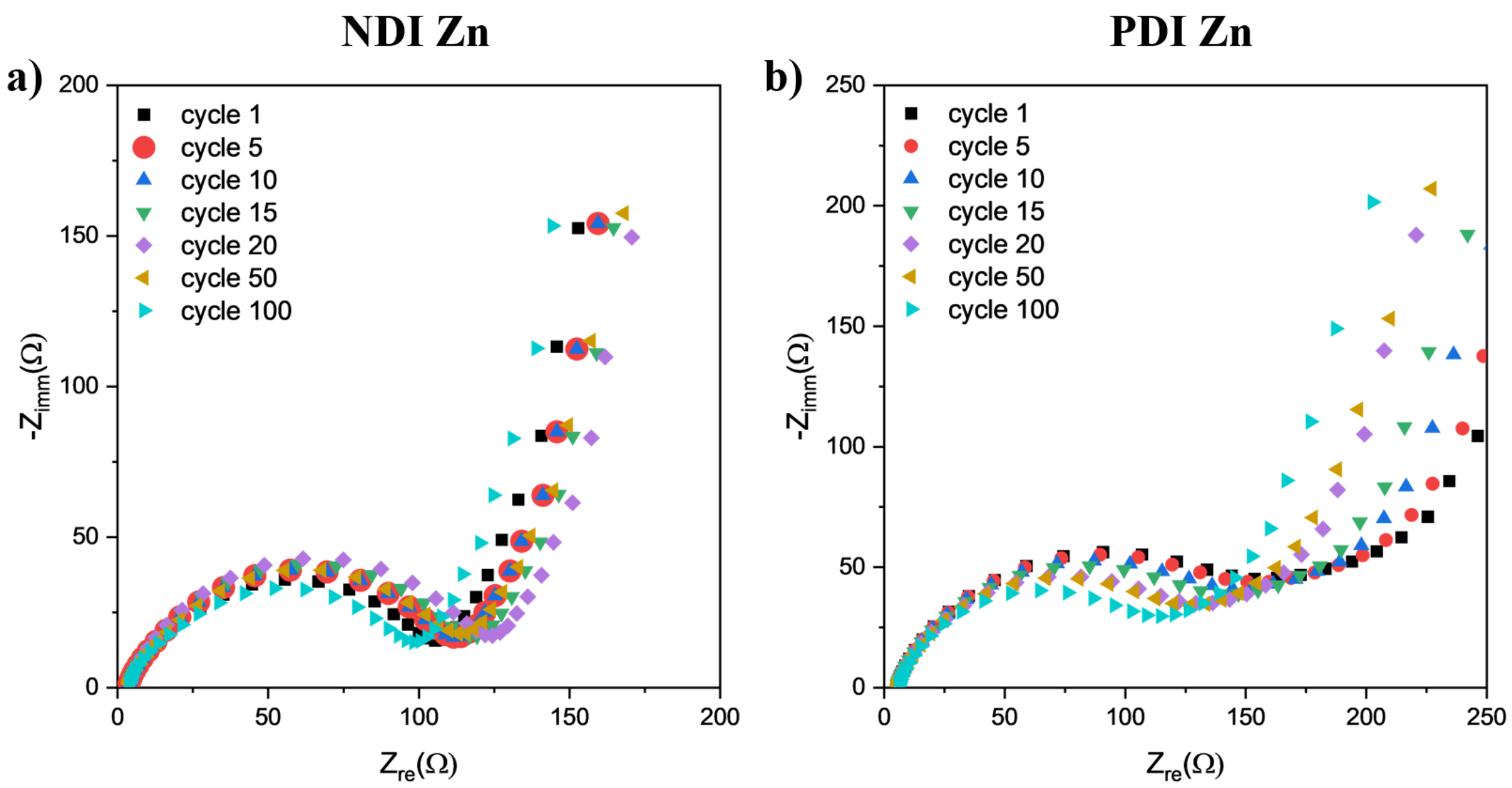

Figure S12. Nyquist plots obtained by EIS of (a) **Zn–NDI** and (b) **Zn–PDI** electrodes at selected cycle numbers (1, 5, 10, 15, 20, 50, and 100).

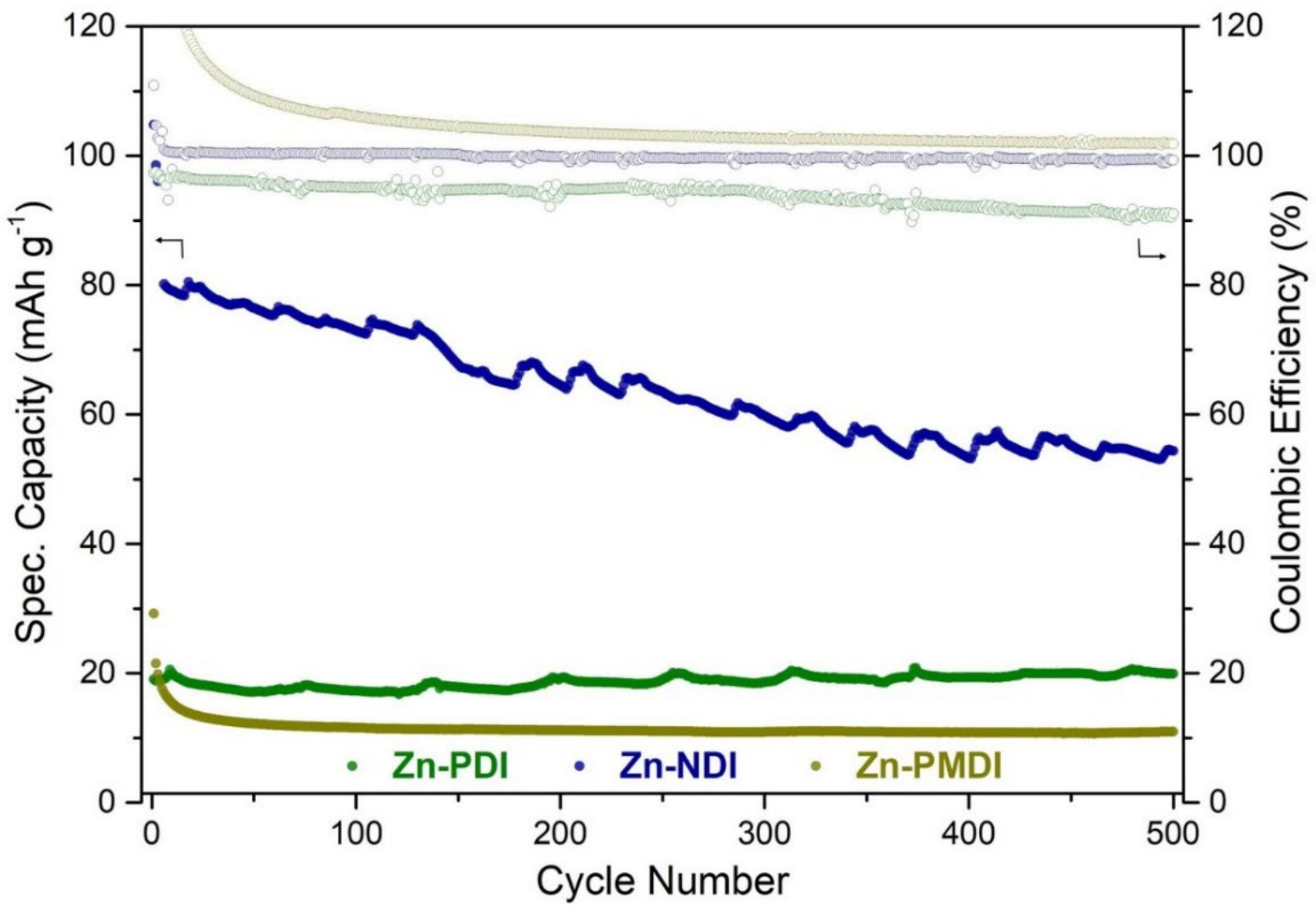

Figure S13. Cycling stability and Coulombic efficiency at a C-rate of 2 C within a potential window of 1.4–3.5 V vs. Li/Li^+ of **Zn-XDI MOFs** in Li-ion batteries with 1 M $LiPF_6$ in EC:DEC (1:1 vol) as electrolyte.

Table S1. Highest specific capacity (rate) and capacity retention for **Zn-NDI**, **Zn-PDI**, and representative NDI- and PDI-based molecular and polymeric materials.

**Compound**	**Highest capacity (rate)**	**Capacity retention (%)**	**Reference**
**Zn-NDI**	110 mAh g^{-1} (0.5 C)	98 (500 cycles)	**This work**
**PNDI-T2 (P46) (polymer)**	51.1 mAh g^{-1} (0.2 C)	95 (2000 cycles)	D. Kim, et al., **2025**, *631*, 236208.
**Ni-NDISA (MOF)**	82 mAh g^{-1} (10 mA g^{-1})	89.7 (200 cycles)	J. Lv, et al., *ACS Appl. Energy Mater.*, **2024**, *7*, 6342-6348.
**PNDI-EG (polymer)**	97 mAh g^{-1} (0.5 C)	81 (1000 cycles)	R. Matsidik, et al., *Macromol. Rapid Commun.*, **2025**, *46*, 2401121.
**DTP-ANDI-COF (COF)**	69 mAh g^{-1} (2.4 C)	100 (700 cycles)	F. Xu, et al., *Sci. Rep.*, **2015**, *5*, 8225.
**NDI (molecule)**	140.4 mAh g^{-1} (50 mA g^{-1})	97 (6000 cycles)	J. Chen et al., *ACS Appl. Mater. Interfaces*, **2022**, *14*, 44330-44337.
**Zn-PDI**	75 mAh g^{-1} (0.5 C)	87 (500 cycles)	**This work**
**PDI–Tc (3D COF)**	75.9 mAh g^{-1} (0.05 C)	80.2 (500 cycles)	T. N. Schon, et al., *ACS App. Mater. Interfaces*, **2017**, *9*, 15631-15637.
**PDI-3AT (molecule)**	92.7 mAh g^{-1} (50 mA g^{-1})	77 (1000 cycles)	H. Seong, et al., *ACS Applied Materials & Interfaces*, **2023**, *15*, 58451-58461.

4. <u>Charge storage mechanism</u>

The lithiation mechanism was investigated by cyclic voltammetry (CV) at various scan rates for both **Zn-NDI** and **Zn-PDI**. The analysis employed the power-law relationship (**Equation S1**):

$$I = av^b$$

Where I represent the current peak, v the scan rate, and a and b the two unknown values to find.

Then the relationship between current (I) and the scan rate (v) was linearized in logarithmic form using **Equation S2**:

$$log\ (I) = b \cdot log\ (v) + log(a)$$

The exponent b provides mechanistic insight: values close to 0.5 indicate diffusion-controlled process, whereas values approaching 1.0 suggest a surface-controlled, pseudocapacitive behaviour. The value of b was calculated through a linear fit of the two plots.[6]

As shown in **Figure S14a** and **S14b**, **Zn-NDI** exhibits lower b values, pointing to a more diffusion-limited charge storage process. Conversely, the **Zn-PDI** exhibits values near unity, highlighting a predominant capacitive contribution.

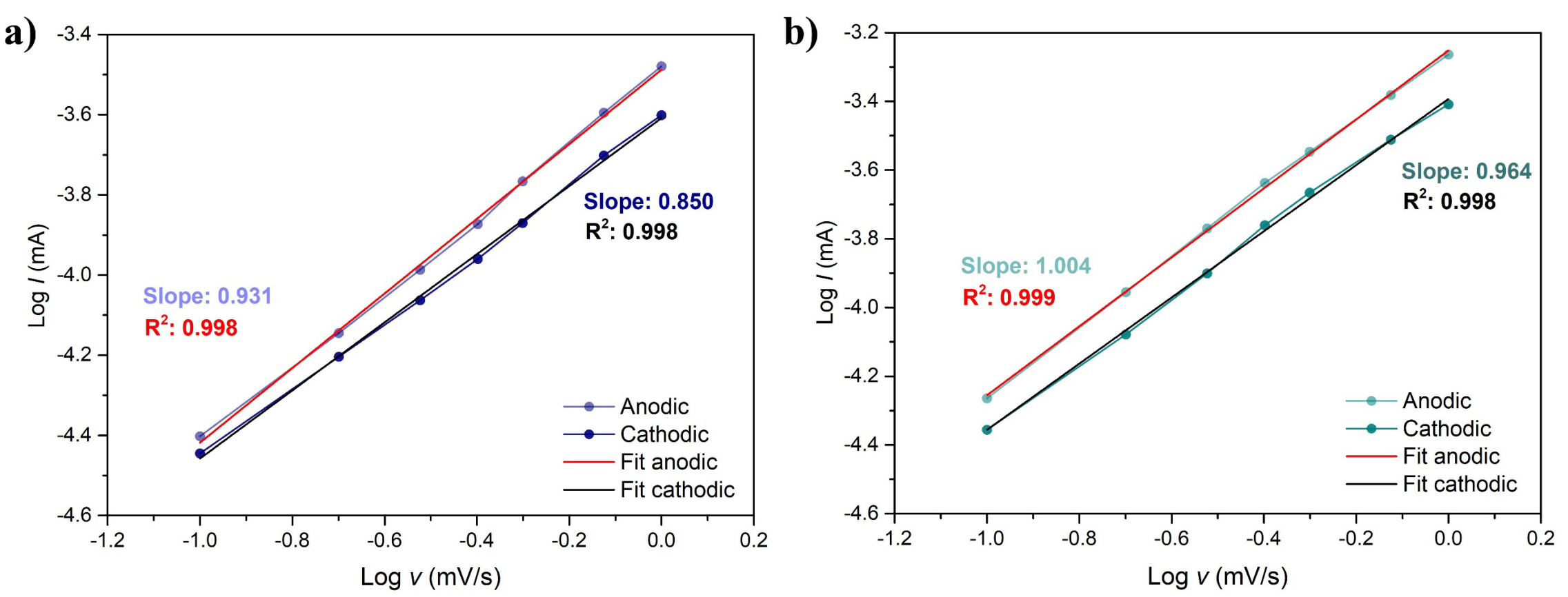

Figure S14. Logarithmic scale of I *vs* logarithmic scale of the scan rate and their linear fit for the cathodic and anodic peaks of (a) **Zn-NDI** and (b) **Zn-PDI**.

To complement this quantitative assessment, a quantitative analysis based on Dunn's method was conducted to separate capacitive and diffusion-controlled contributions in the two materials.[6] In this method, the current response is analysed at fixed potentials, where the capacitive current scales linearly with the scan rates (v), while the diffusion-controlled current scales with the square root of the scan rate ($v^{1/2}$). This relationship is described by **Equation S3**:

$$i_{total} = i_{capacitive} + i_{diffusive}$$

This expression can be rearranged as shown in **Equation S4**:

$$\frac{i(v)}{v^{1/2}} = k_1 v^{1/2} + k_2$$

Here, k_1 (surface-controlled coefficient) and k_2 (diffusion-controlled coefficient) were obtained from the slope and intercept of the linear fits shown in **Figure S14a** and **S14b**, with the corresponding values summarized in **Table S2.**

Table S2. Values of k_1 and k_2 obtained applying Dunn's method through the linear fit shown in plots of **Figure S14a** and **S14b** for **Zn-NDI** and **Zn-PDI**.

Sample	Peak	k_1	k_2
**Zn-NDI**	Cathodic	-0.205	-0.0472
	Anodic	0.308	0.0228
**Zn-PDI**	Cathodic	-0.379	-0.0241
	Anodic	0.547	0.00724

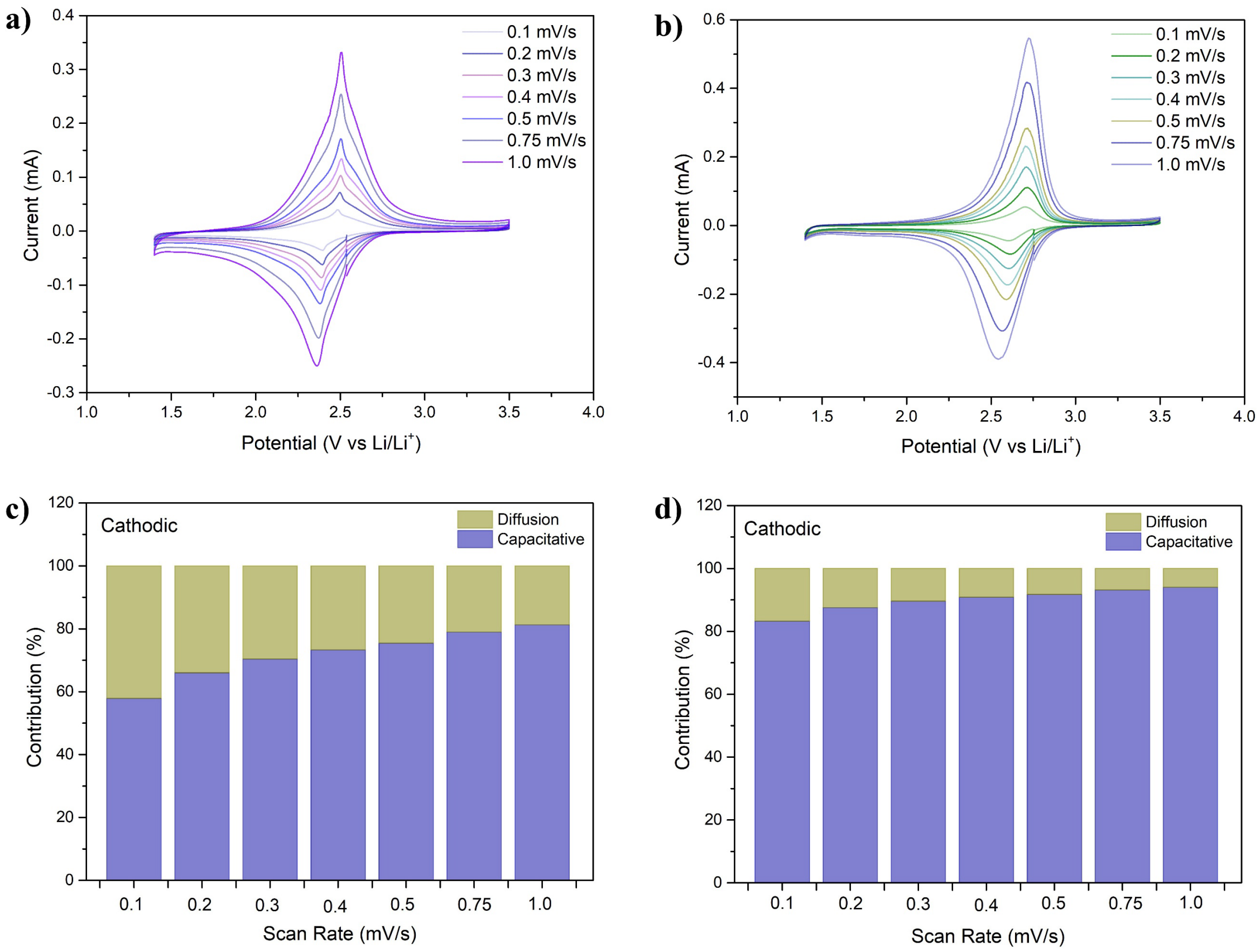

Figure S15. Intensity/square root of the scan rate vs the square root of the scan rate and their linear fit for the cathodic and anodic peaks of (a) **Zn-NDI** and (b) **Zn-PDI**. Capacitive and diffusive contributions in percentage (%) obtained for anodic peak of (a) **Zn-NDI** and (b) **Zn-PDI**.

Randles-Ševčik was used to calculate the diffusion coefficients of lithium ions in **Zn-XDI** MOFs according to **Equation S5**:[6]

$$I_p = (2.69 \times 10^5) n^{3/2} A C D^{1/2} v^{1/2}$$

Table S3. Diffusion coefficient of **Zn-NDI** and **Zn-PDI** calculated for cathodic and anodic peaks.

Sample	Peak	D_{Li^+} (cm²/s)
**Zn-NDI**	Cathodic	8.57×10^{-11}
	Anodic	1.57×10^{-10}
**Zn-PDI**	Cathodic	5.62×10^{-10}
	Anodic	1.09×10^{-9}

5. *Post-mortem* studies

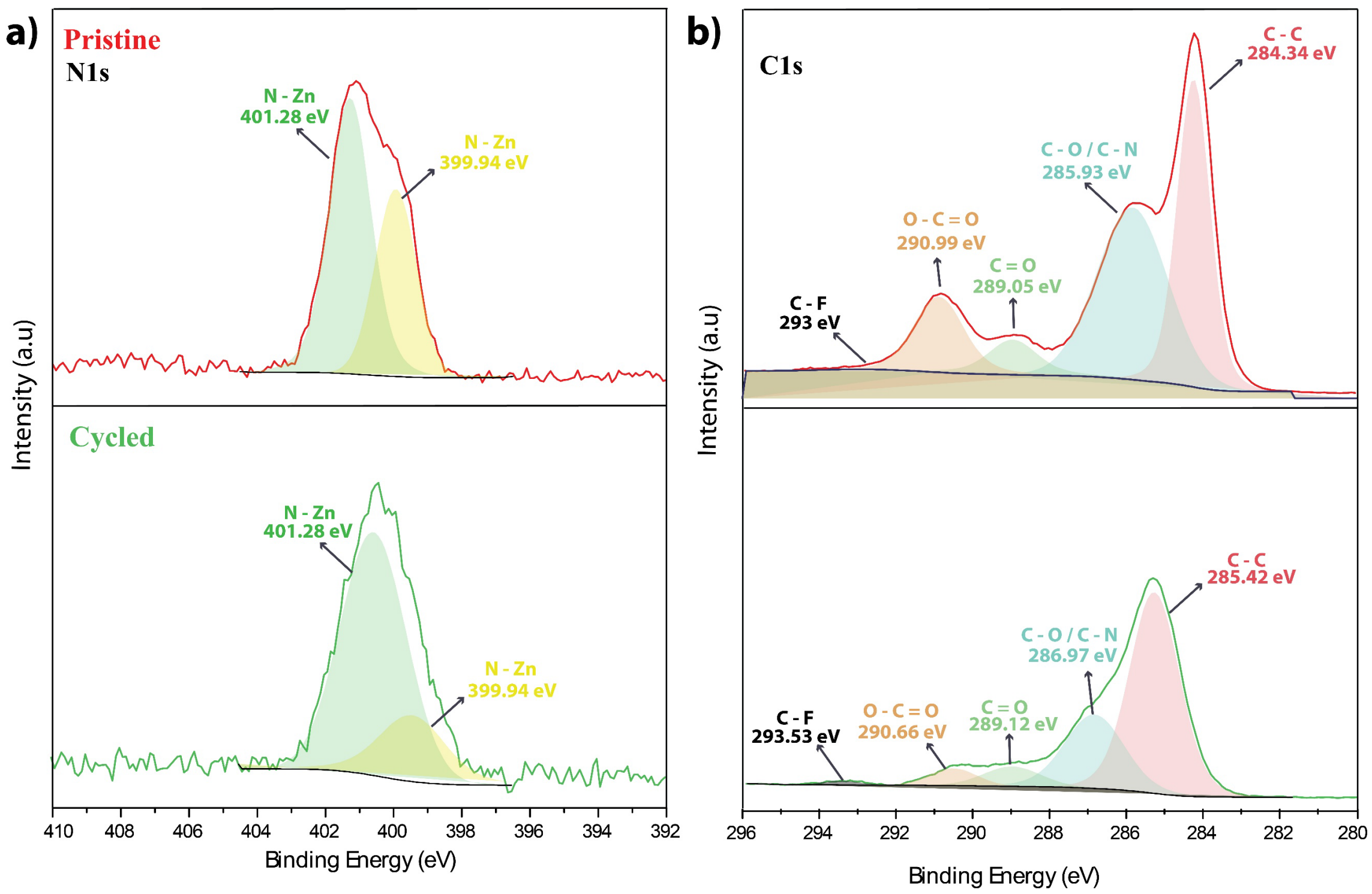

Figure S16. *Ex-situ* XPS spectra of **Zn-NDI** MOF electrodes (pristine and after 1000 cycles). (a) N1s and (b) C1s.

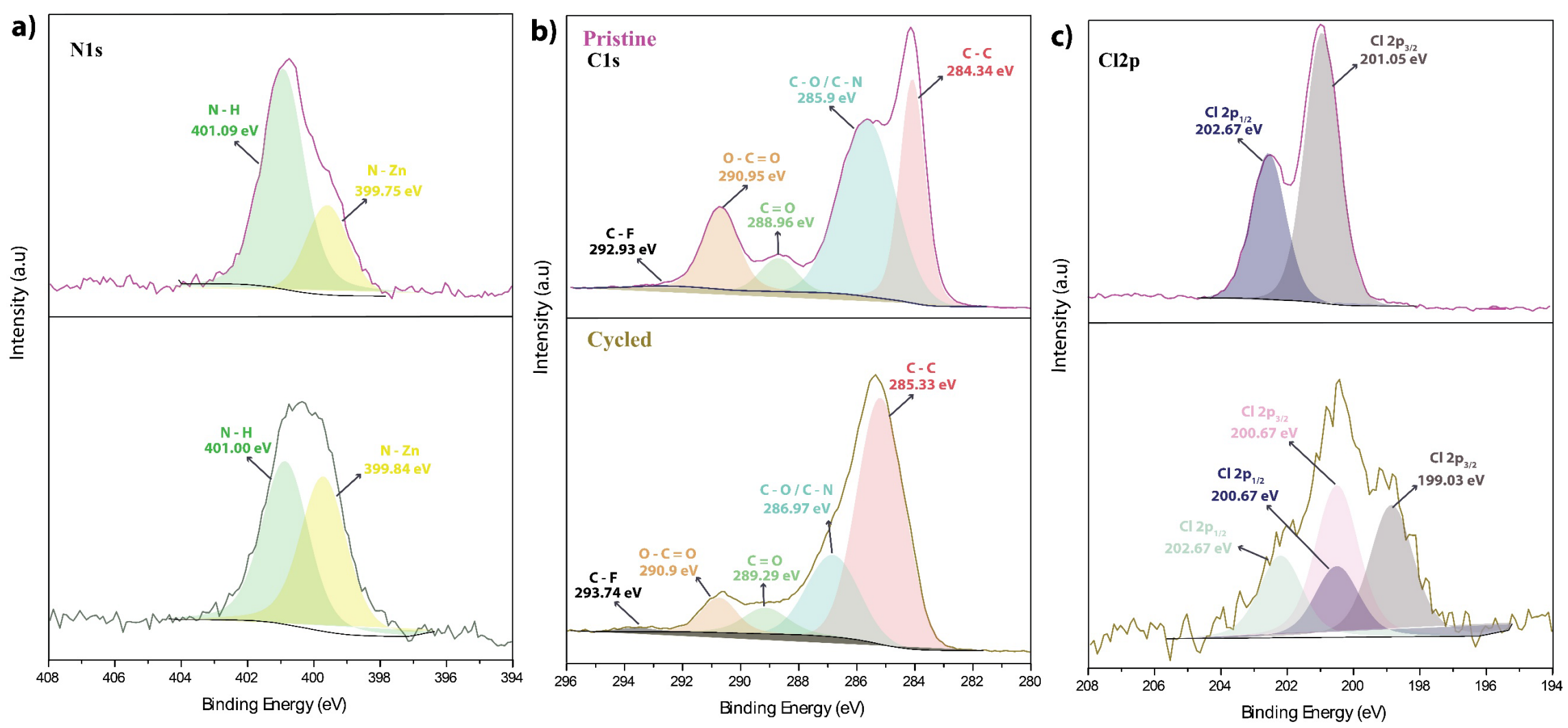

Figure S17. *Ex-situ* XPS spectra of **Zn-PDI** MOF electrodes (pristine and after 1000 cycles). (a) N1s, (b) C1s and (c) Cl2p.

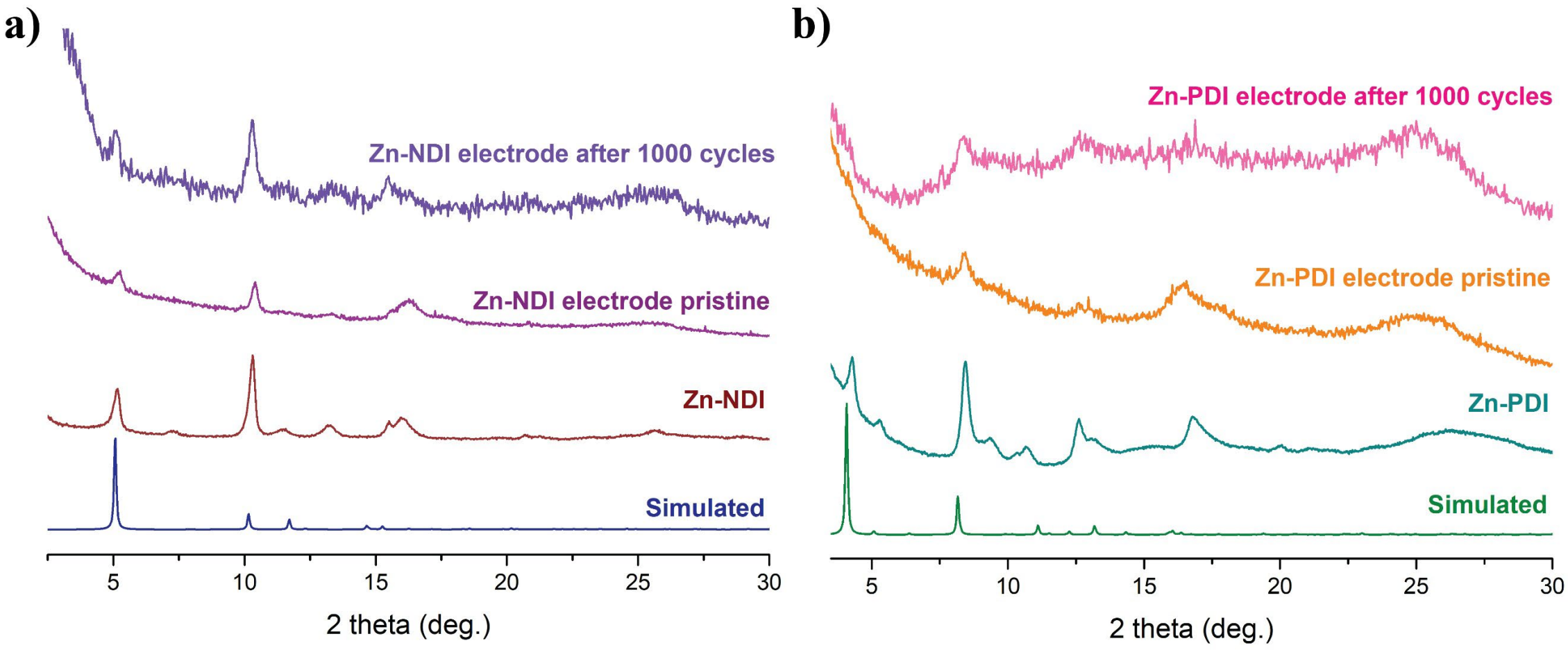

Figure S18. *Ex-situ* PXRD patterns, comparing the as-synthesized material with the electrodes before and after cycling of (a) **Zn-NDI** and (b) **Zn-PDI** MOF.

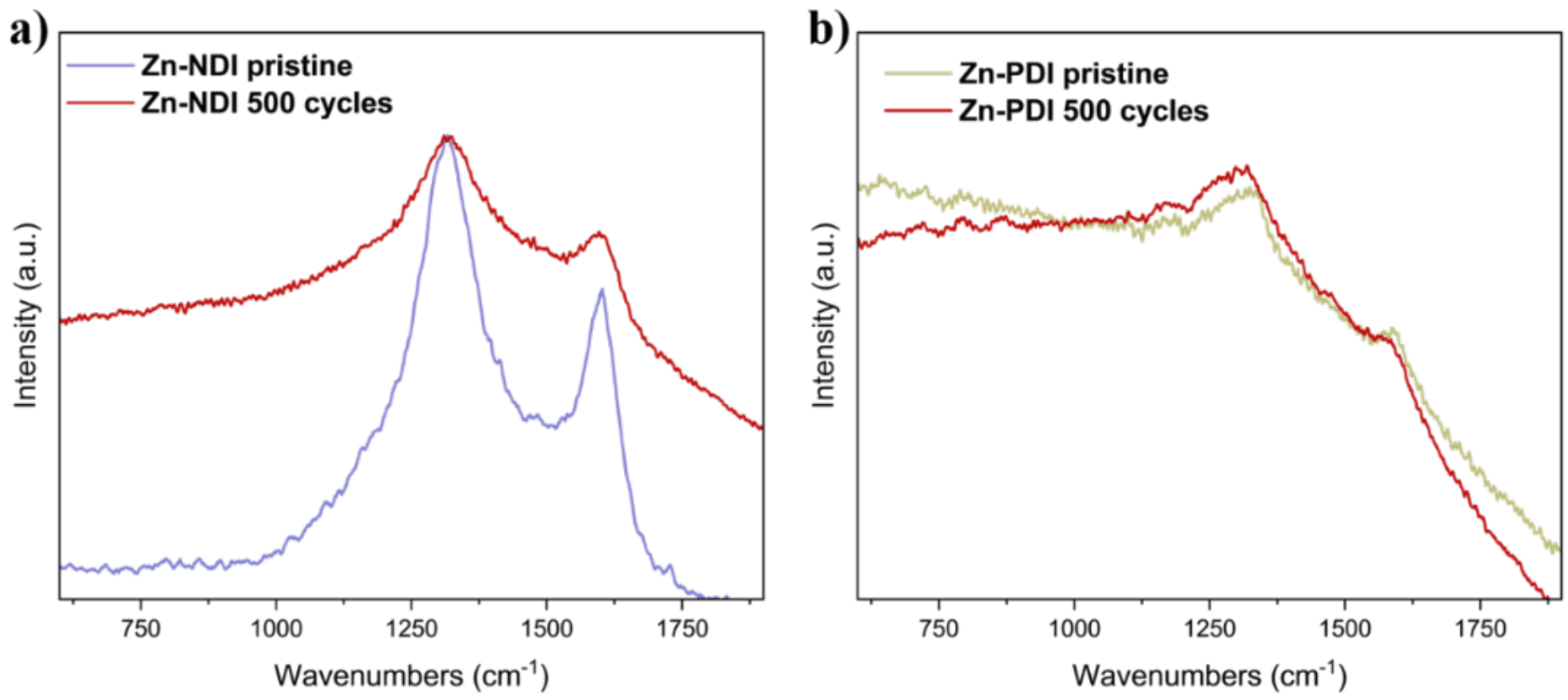

Figure S19. Raman spectra of pristine and cycled (500 cycles) a) **Zn-NDI** and b) **Zn-PDI** electrodes.

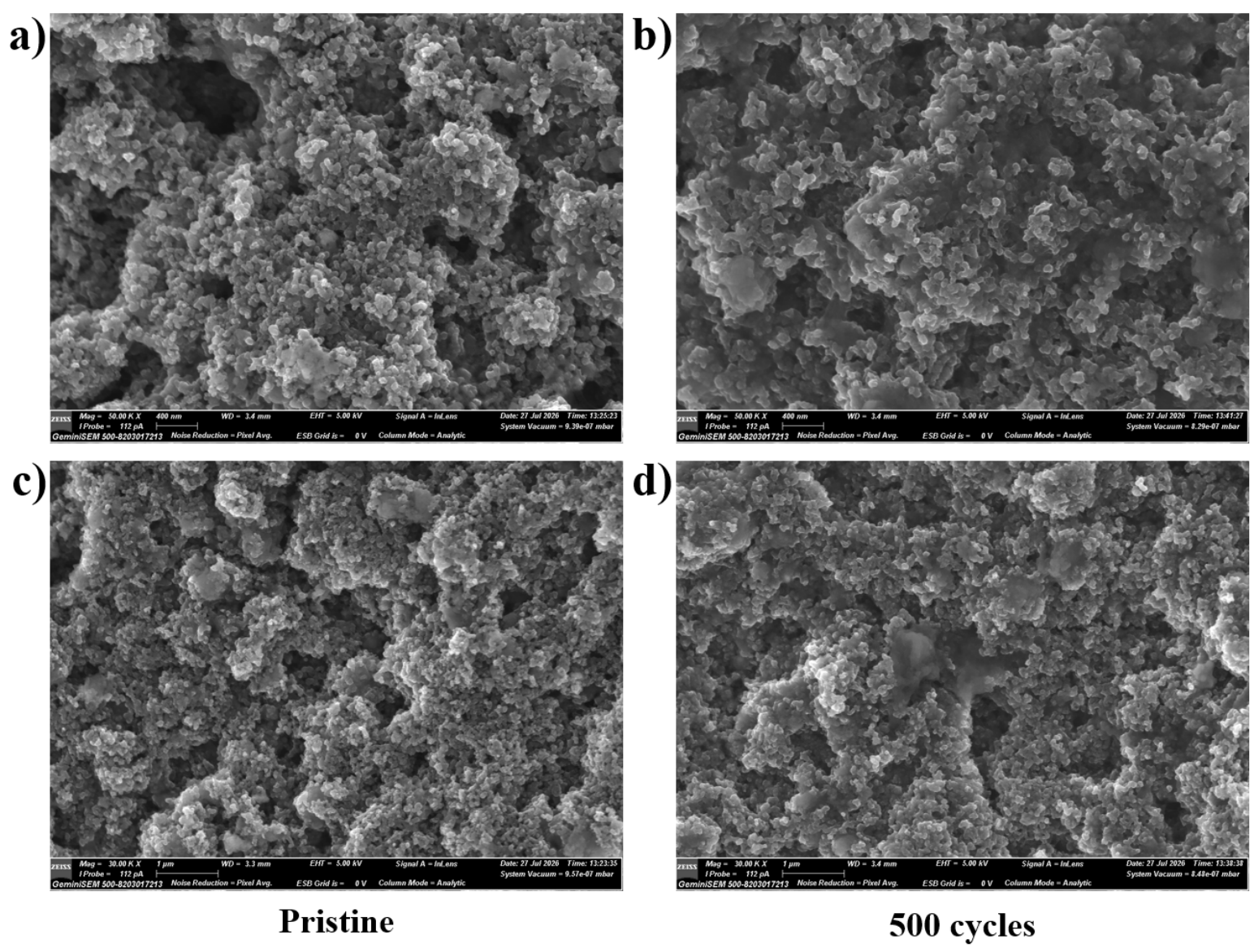

Figure S20. SEM images of pristine (a and c) and cycled (500 cycles) (b and d) of **Zn-NDI** electrodes.

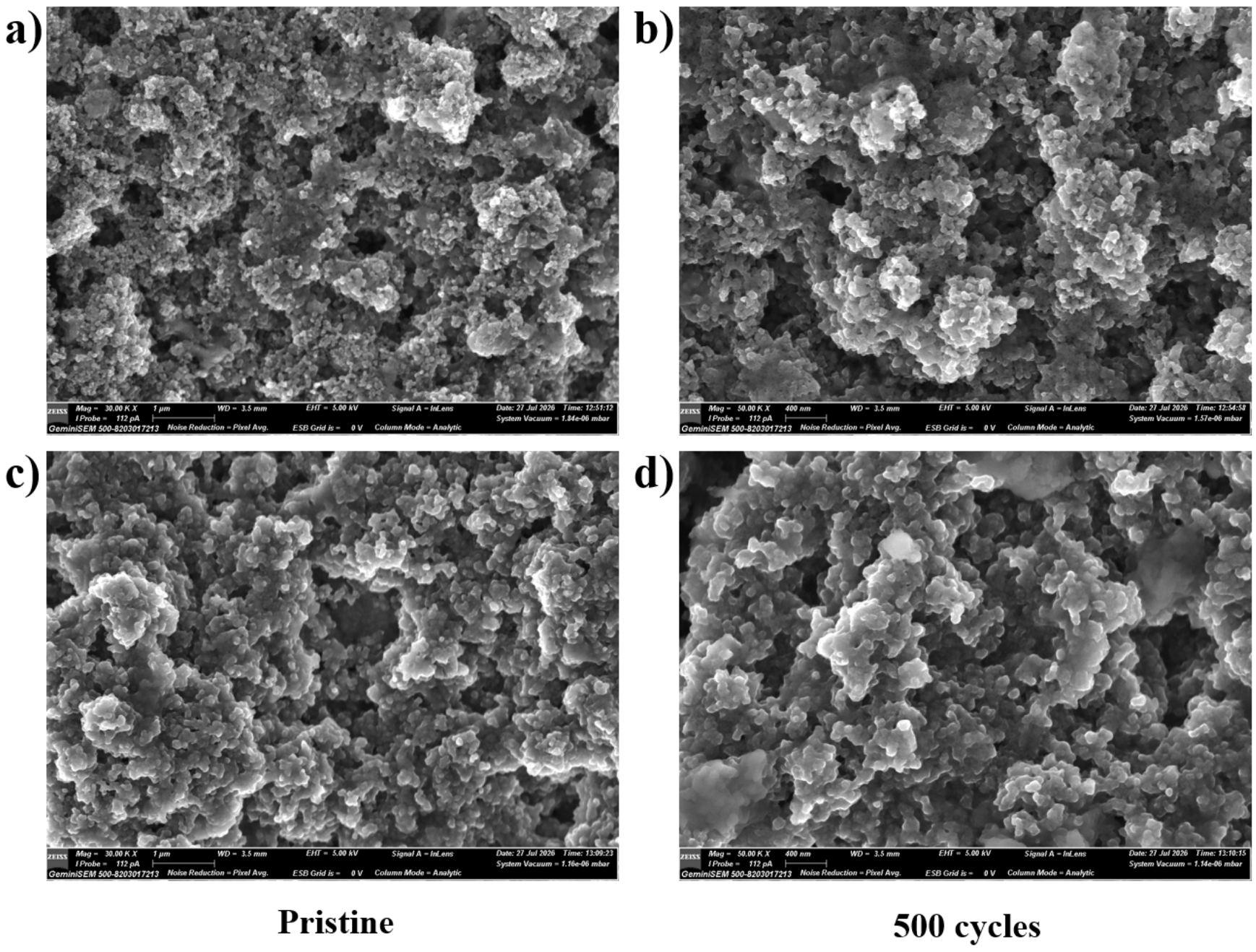

Figure S21. SEM images of pristine (a and c) and cycled (500 cycles) (b and d) **Zn-PDI** electrodes.

6. Computational details

Theoretical calculations were performed by means of the density functional theory (DFT). Geometry optimization and frequencies of the three linkers (PMDI, NDI and PDI) were computed with the Gaussian-16.a03 program suite,[7] using the hybrid HSE06 functional[8] with the default ω = 0.11 for short range Hartree-Fock and ω = 0.189 for the long-range DFT exchange-correlation energy, and a Pople's 6-31G(d,p) doubly polarized split-valence basis set.[9] First and second electron affinities were computed including solvent effects by means of the SMD continuum solvent model with solvent parameters simulating DOL/DME (1:1, v/v) mixture.[10]

Periodic boundary conditions calculations were performed for the **Zn-XDI** crystal structures using the PBEsol functional[11] through the FHI-aims software,[12] to relax the atomic positions and unit cell parameters, together with the Tkatchenko-Scheffler van der Waals correction.[13] Achieving convergence is such relaxations in a reasonable computational time required to perform a pre-relaxation step with the "light" settings of the numerical atom-centered orbital basis set, and then refine the structure with the "intermediate" settings, as implemented in FHI-aims. Single-point electronic structure calculations were performed for the minimum-energy crystal geometries at the hybrid HSE06 level to explore the reciprocal space. The choice of the *k*-grid was 3×3×6 for Zn-PMDI, 3×5×12 for Zn-NDI, and 3×3×12 for Zn-PDI, and the Gaussian smearing method was applied to compute the species-projected density of states. The reciprocal space was explored along the path Γ–X–S–Y–Γ–Z–U–R–T–Z. Finally, relativistic effects were included through the scalar zeroth-order regular approximation to the Dirac equation (ZORA) as implemented in FHI-aims. Molecular structures, molecular orbitals, electrostatic potential maps and spin density representations were plotted by means of the Chemcraft software.[14] The topology of the frontier crystal orbitals associated to the valence band maximum and conduction band minimum were plotted through the VESTA software.[15]

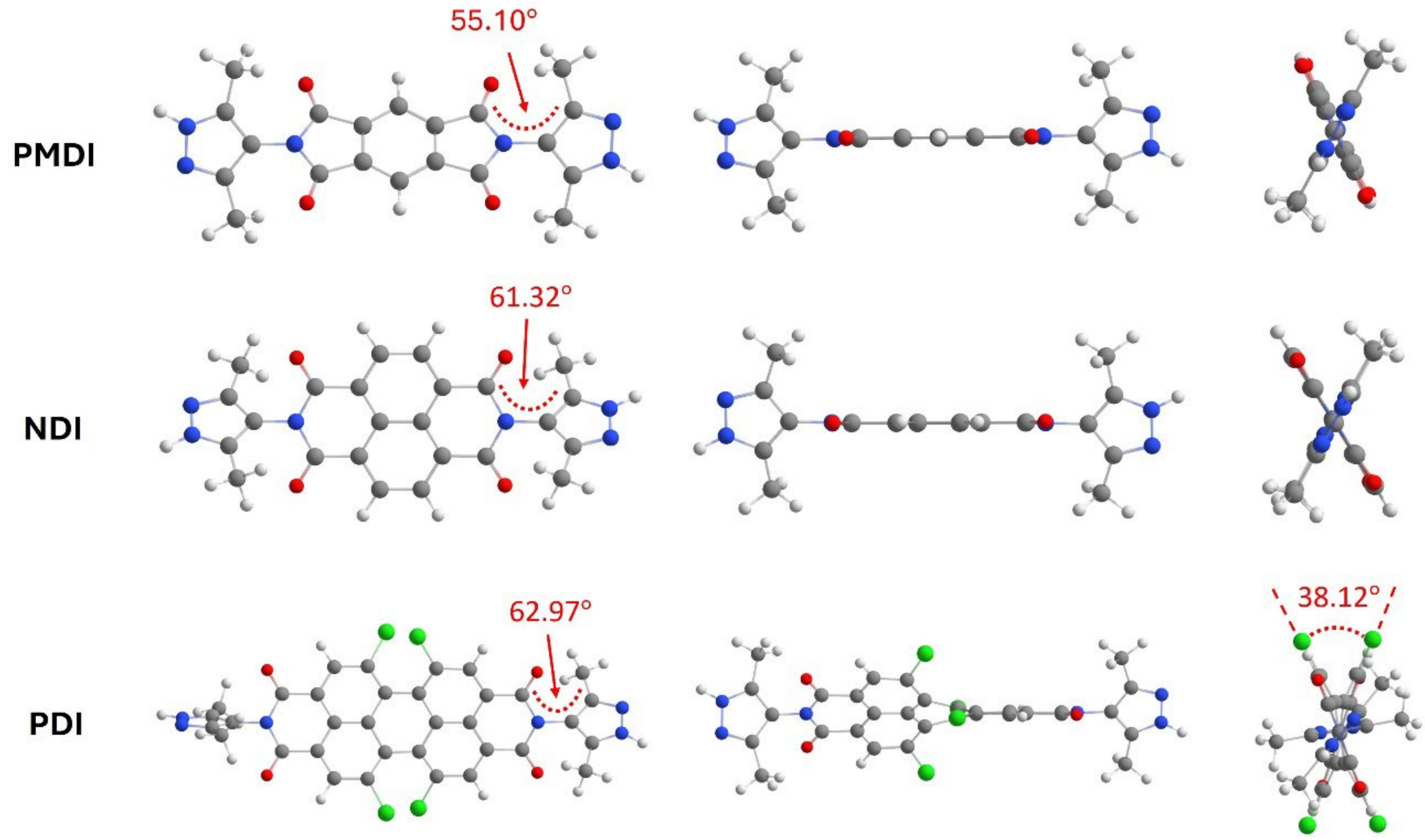

Figure S22. Top (left), front (middle) and side (right) views of the minimum-energy structure of the organic ligands calculated at the HSE06/6-31G(d,p) level of theory.

Table S4. Effect of the π-core twisting and the presence of the Cl substituents on PDI.

	**LUMO energy (eV)**	**Relative energy (eV)**
PDI (with Cl substituents)	-4.07	-
PDI with H instead of Cl atoms at the geometry of PDI with Cl atoms	-3.73	0.90
PDI with H instead of Cl atoms after fully optimized	-3.79	0.00

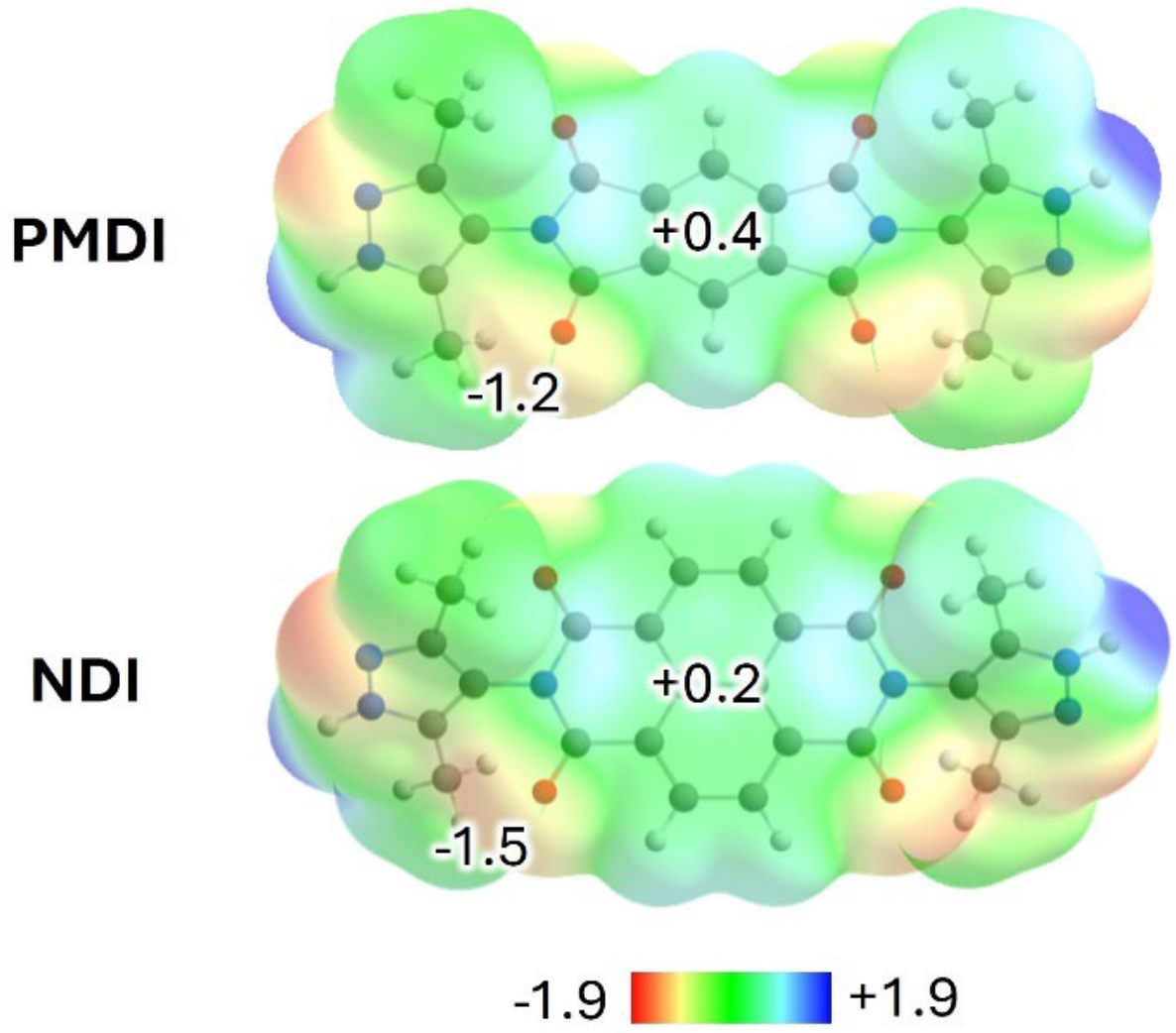

Figure S23. Molecular electrostatic potential maps of neutral ligands mapped onto the electron density isosurface (isovalue of 0.001 a.u.). The colour scale and relevant energy values of the surface are given in eV.

Table S5. Net charge (in *e*) retained by the lithium atom in singly lithiated Li-XDI species computed by Mulliken and Atomic Polar Tensor (APT) charge models at the HSE06/6-31G(d,p) level in vacuum.

	Li-PMDI	Li-NDI	Li-PDI
**Mulliken**	+0.511	+0.507	+0.518
**APT**	+0.861	+0.839	+0.912

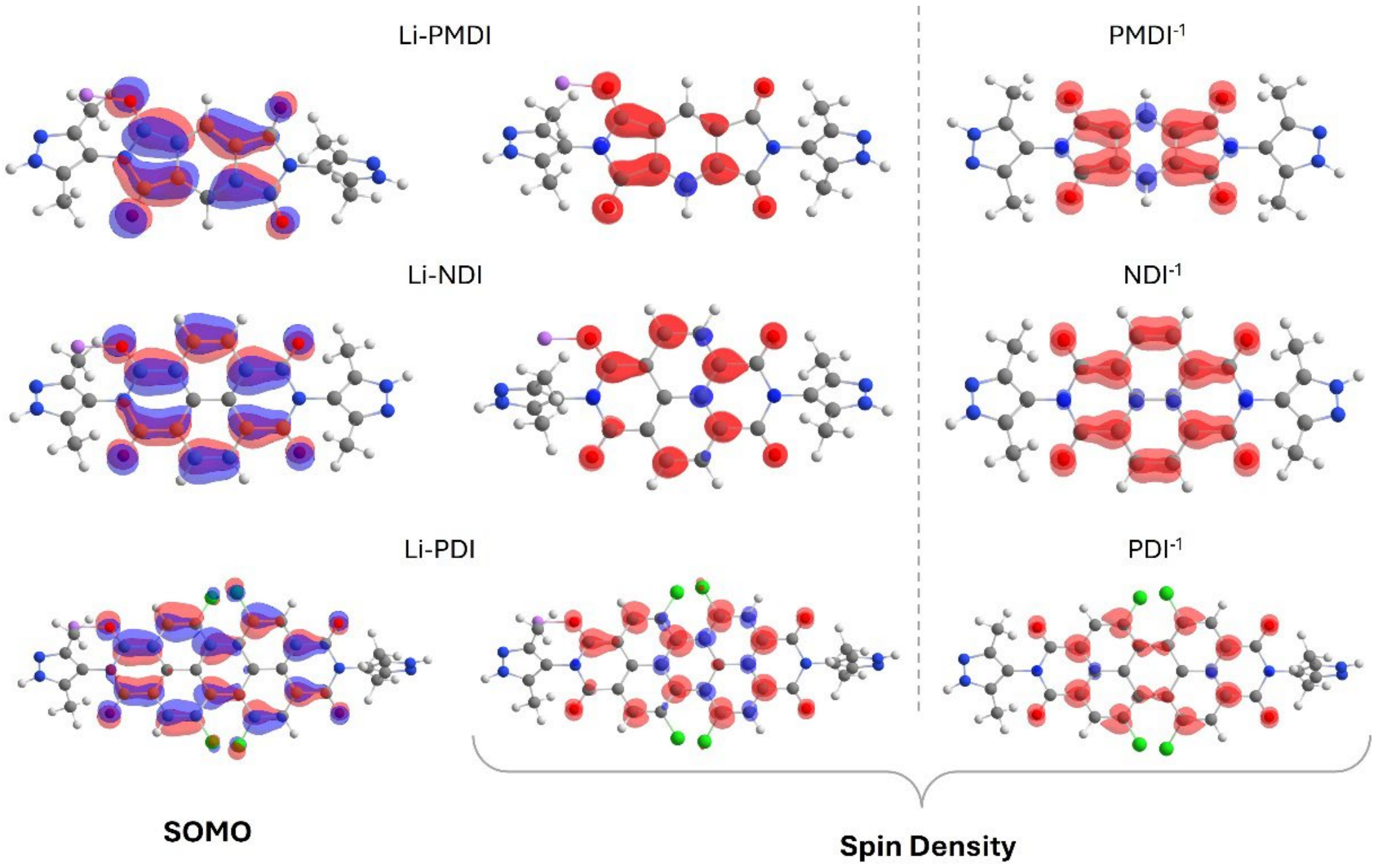

Figure S24. Comparison of anionic ligand species spin density (right) with Li-XDI spin density (middle) and Li-XDI singly-occupied molecular orbital (SOMO) (left) calculated at the HSE06/6-31G(d,p) level in vacuum. SOMO isovalue of 0.03 a.u. and spin density isovalue of 0.003 a.u.

Table S6. Binding energy of the first and second Li-XDI complexation calculated at the HSE06/6-31G(d,p)+SMD level of theory.

	**Binding energy (eV)**		
	PMDI	NDI	PDI
**(Li-XDI) - (Li^0+XDI^0)**	-2.54	-2.83	-3.07
**(2Li-XDI) - (2Li^0+XDI^0)**	-4.79	-5.47	-6.07
**(Li-XDI) - (Li^{+1}+XDI^{-1})**	-1.71	-1.77	-1.60
**(2Li -XDI) - (2Li^{+1}+XDI^{-2})**	-4.58	-4.56	-3.97

Table S7. Relative energy (in eV) of second lithiation onto XDI ligand, calculated at the HSE06/6-31G(d,p) level of theory in vacuum.

**Lithiation positions**	**Singlet**	**Triplet**	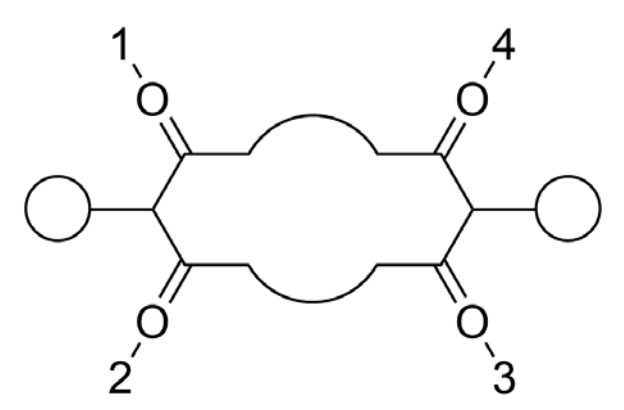
1 and 2	0.22	1.04	
1 and 3	0.00	1.11	
1 and 4	0.14	0.76	

Table S8. Crystal structure parameters before (initial; experimental structure) and after (relaxed) full lattice and atomic position optimization at the PBEsol level of theory. The difference (in %) between the two structures is also indicated (Δ).

	PMDI			NDI			PDI		
**Cell parameters**	**Initial**	**Relaxed**	**Δ (%)**	**Initial**	**Relaxed**	**Δ (%)**	**Initial**	**Relaxed**	**Δ (%)**
a (Å)	21.476	27.059	26	22.247	27.693	24	27.663	34.599	25
b (Å)	26.999	20.47	-24	27.968	20.765	-26	34.778	25.816	-26
c (Å)	8.017	6.891	-14	8.384	6.882	-18	8.559	6.743	-21
α (º)	90.0	90.1	0	90.0	90.0	0	90.0	90.3	0
β (º)	90.0	86.5	-4	90.0	85.4	-5	90.0	84.3	-6
γ (º)	90.0	90.0	0	90.0	90.0	0	90.0	90.0	0
Volume (Å^3)	4648	3810	-18	5216	3945	-24	8234	5994	-27

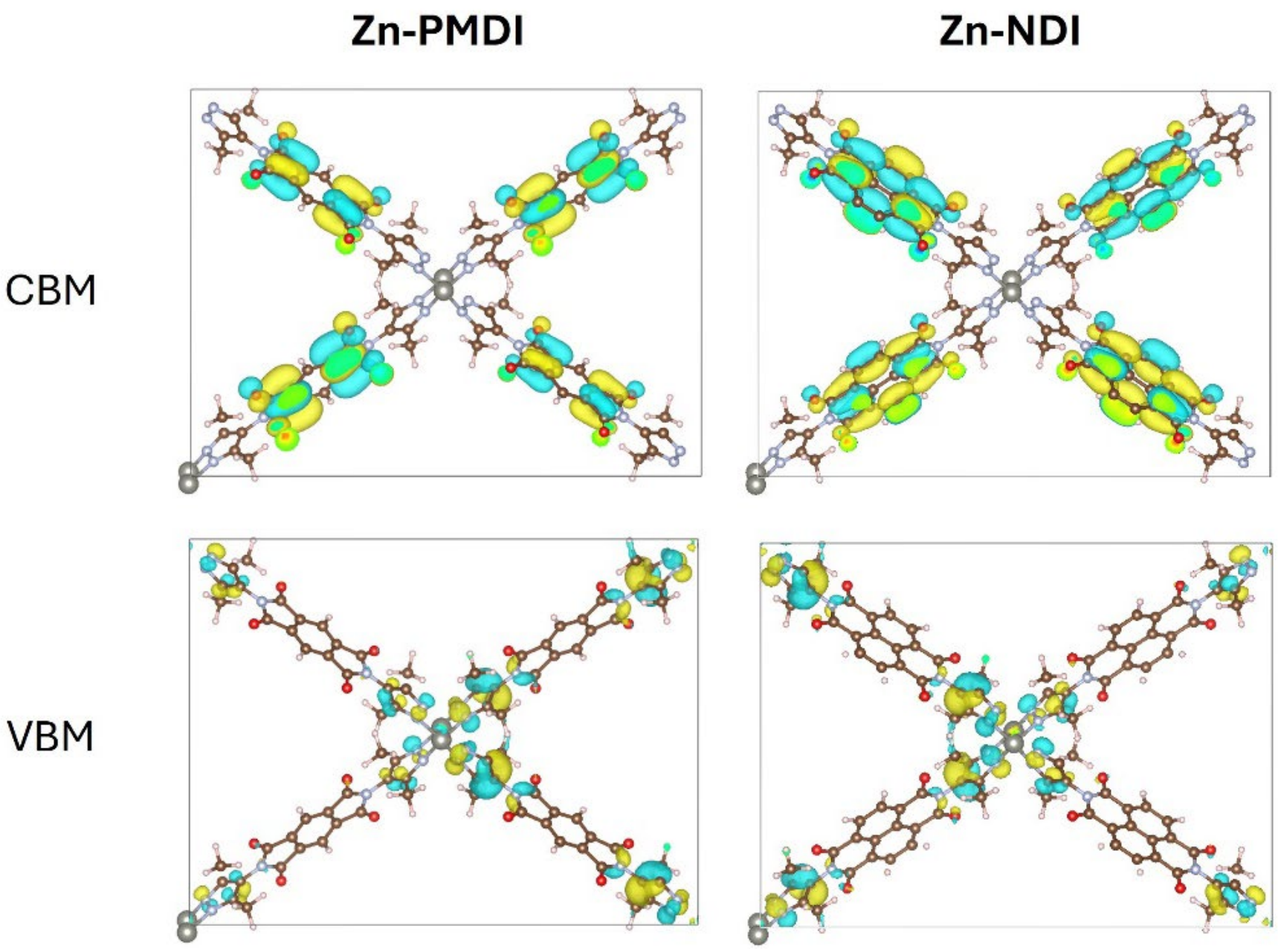

Figure S25. Topology of the crystal orbitals corresponding to the valence band maximum (VBM) and conduction band minimum (CBM) calculated for **Zn-PMDI** and **Zn-NDI** at the HSE06 level of theory.

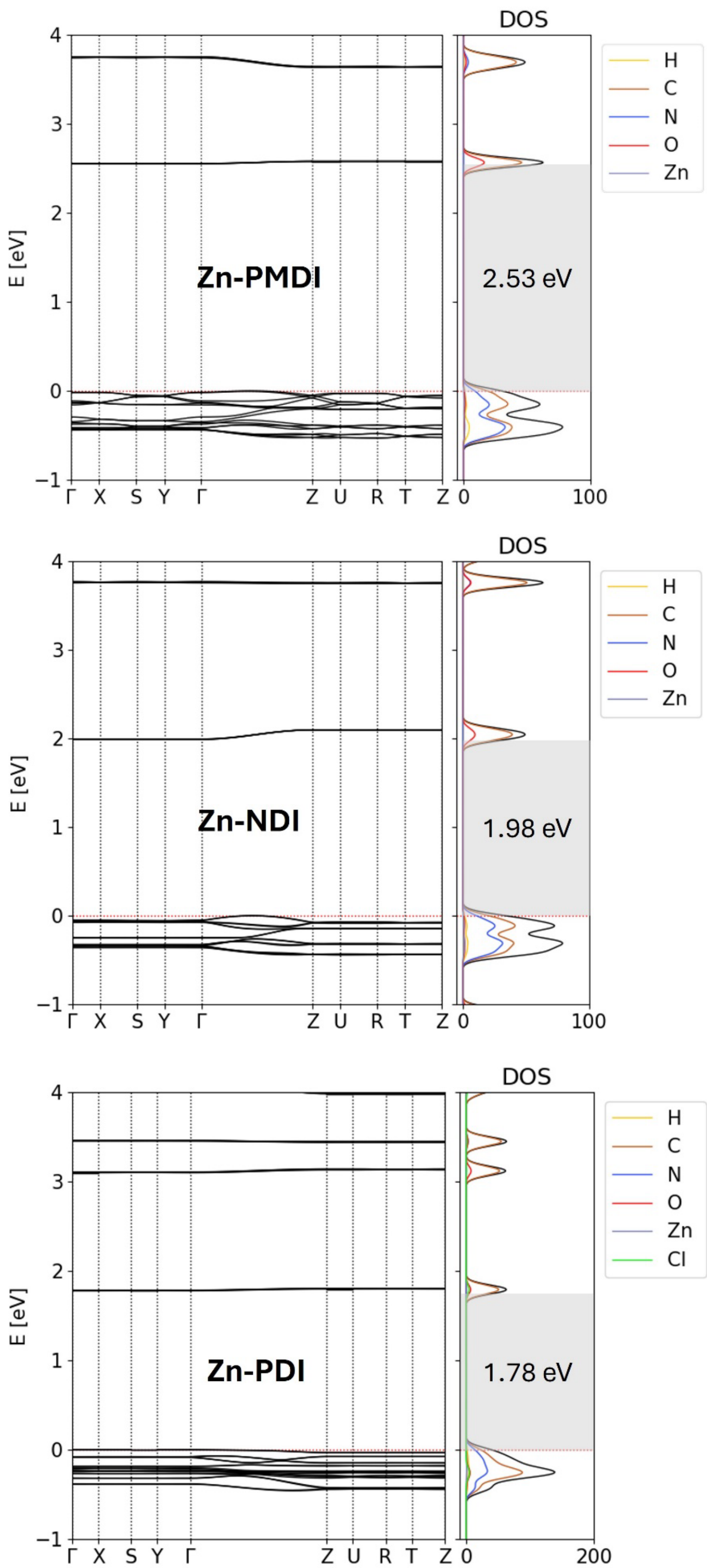

Figure S26. Electronic band structure and density of states calculated at the HSE06 level of theory for the **Zn-XDI** family of MOFs. Fermi level energy is set to zero at the top of the valence band.

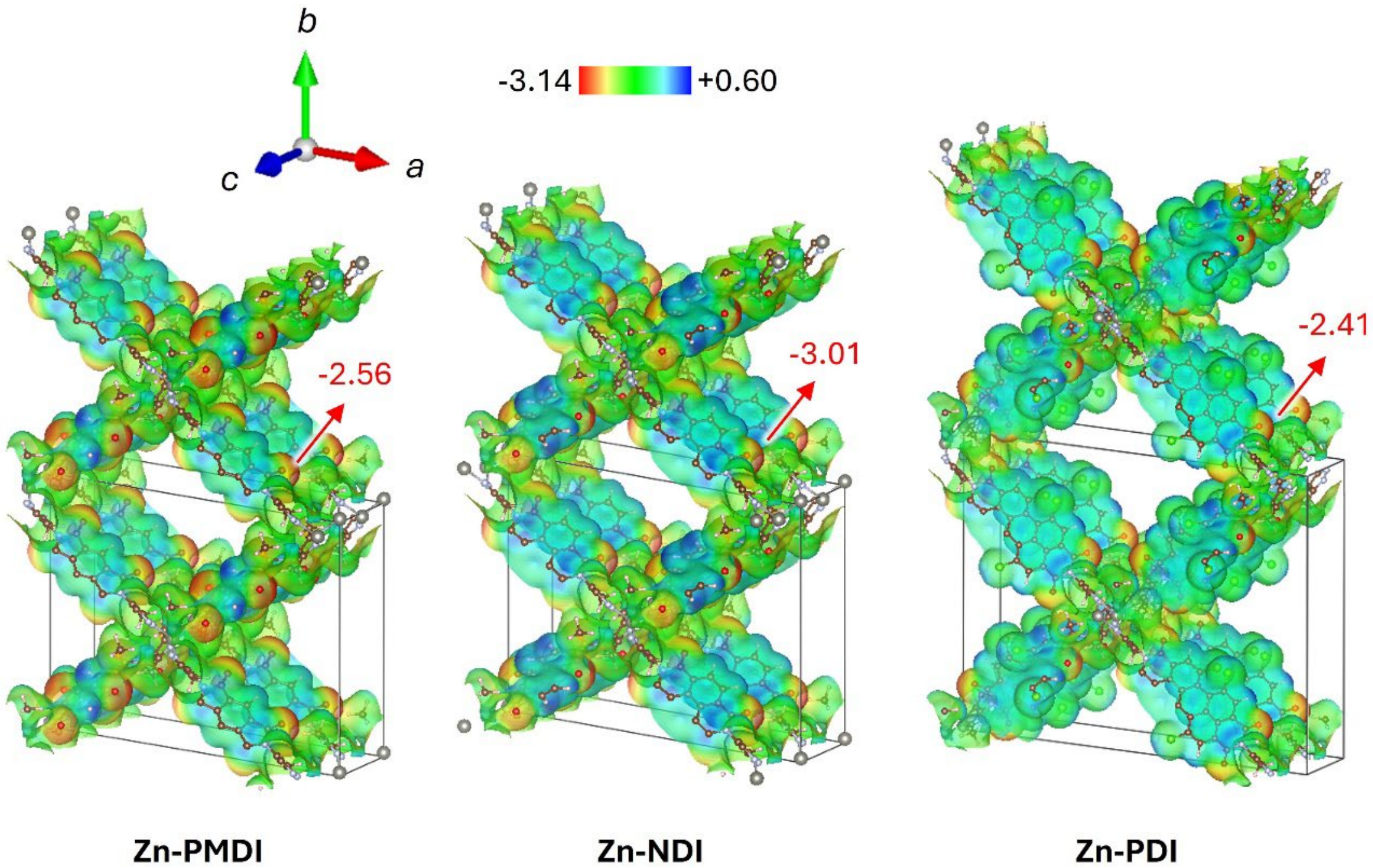

Figure S27. Hartree potential mapped onto the electron density isosurface (isovalue of 0.001 a.u.) calculated at the HSE06 level of theory for the **Zn-XDI** family of MOFs. The colour scale and relevant energy values of the surface are given in eV.

7. References

(1) Kumar, A.; Li, J.; Inge, A. K.; Ott, S. Electrochromism in Isoreticular Metal–Organic Framework Thin Films with Record High Coloration Efficiency. *ACS Nano* **2023**, *17*, 21595–21603. https://doi.org/10.1021/acsnano.3c06621.

(2) Jiang, J.-X.; Su, F.; Trewin, A.; Wood, C. D.; Niu, H.; Jones, J. T. A.; Khimyak, Y. Z.; Cooper, A. I. Synthetic Control of the Pore Dimension and Surface Area in Conjugated Microporous Polymer and Copolymer Networks. *J. Am. Chem. Soc.* **2008**, *130*, 7710–7720. https://doi.org/10.1021/ja8010176.

(3) Eddaoudi, M.; Kim, J.; Rosi, N.; Vodak, D.; Wachter, J.; O'Keeffe, M.; Yaghi, O. M. Systematic Design of Pore Size and Functionality in Isoreticular MOFs and Their Application in Methane Storage. *Science* **2002**, *295*, 469–472. https://doi.org/10.1126/science.1067208.

(4) Yao, Y.; Zhao, X.; Chang, G.; Yang, X.; Chen, B. Hierarchically Porous Metal–Organic Frameworks: Synthetic Strategies and Applications. *Small Struct.* **2023**, *4*. https://doi.org/10.1002/sstr.202200187.

(5) Furukawa, H.; Cordova, K. E.; O'Keeffe, M.; Yaghi, O. M. The Chemistry and Applications of Metal-Organic Frameworks. *Science* **2013**, *341*, 1230444. https://doi.org/10.1126/science.1230444.

(6) Kim, T.; Choi, W.; Shin, H. C.; Choi, J. Y.; Kim, J. M.; Park, M. S.; Yoon, W. S. Applications of Voltammetry in Lithium Ion Battery Research. *J. Electrochem. Sci. Technol*. **2020**, 14–25. https://doi.org/10.33961/jecst.2019.00619.

(7) Frisch, M. J.; Trucks, G. W.; Schlegel, H. B.; Scuseria, G. E.; Robb, M. A.; Cheeseman, J. R.; Scalmani, G.; Barone, V.; Petersson, G. A.; Nakatsuji, H.; Li, X.; Caricato, M.; Marenich, A. V.; Bloino, J.; Janesko, B. G.; Gomperts, R.; Mennucci, B.; Hratchian, H. P.; Ortiz, J. V.; Izmaylov, A. F.; Sonnenberg, J. L.; Williams-Young, D.; Ding, F.; Lipparini, F.; Egidi, F.; Goings, J.; Peng, B.; Petrone, A.; Henderson, T.; Ranasinghe, D.; Zakrzewski, V. G.; Gao, J.; Rega, N.; Zheng, G.; Liang, W.; Hada, M.; Ehara, M.; Toyota, K.; Fukuda, R.; Hasegawa, J.; Ishida, M.; Nakajima, T.; Honda, Y.; Kitao, O.; Nakai, H.; Vreven, T.; Throssell, K.; Montgomery, J. A. Jr.; Peralta, J. E.; Ogliaro, F.; Bearpark, M. J.; Heyd, J. J.; Brothers, E. N.; Kudin, K. N.; Staroverov, V. N.; Keith, T. A.; Kobayashi, R.; Normand, J.; Raghavachari, K.; Rendell, A. P.; Burant, J. C.; Iyengar, S. S.; Tomasi, J.; Cossi, M.; Millam, J. M.; Klene, M.; Adamo, C.; Cammi, R.; Ochterski, J. W.; Martin, R. L.; Morokuma, K.; Farkas, O.; Foresman, J. B.; Fox, D. J. *Gaussian 16*; Fox, D. J. Gaussian, Inc., Wallingford CT, 2016.

(8) Krukau, A. V.; Vydrov, O. A.; Izmaylov, A. F.; Scuseria, G. E. Influence of the Exchange Screening Parameter on the Performance of Screened Hybrid Functionals. *J. Chem. Phys.* **2006**, *125*, 224106. https://doi.org/10.1063/1.2404663.

(9) Hehre, W. J.; Ditchfield, R.; Pople, J. A. Self—Consistent Molecular Orbital Methods. XII. Further Extensions of Gaussian—Type Basis Sets for Use in Molecular Orbital Studies of Organic Molecules. *J. Chem. Phys.* **1972**, *56*, 2257–2261. https://doi.org/10.1063/1.1677527.

(10) Marenich, A. V.; Cramer, C. J.; Truhlar, D. G. Universal Solvation Model Based on Solute Electron Density and on a Continuum Model of the Solvent Defined by the Bulk Dielectric Constant and Atomic Surface Tensions. *J. Phys. Chem. B* **2009**, *113*, 6378–6396. https://doi.org/10.1021/jp810292n.

(11) Perdew, J. P.; Ruzsinszky, A.; Csonka, G. I.; Vydrov, O. A.; Scuseria, G. E.; Constantin, L. A.; Zhou, X.; Burke, K. Restoring the Density-Gradient Expansion for Exchange in Solids and Surfaces. *Phys. Rev. Lett.* **2008**, *100*, 136406. https://doi.org/10.1103/PhysRevLett.100.136406.

(12) Blum, V.; Gehrke, R.; Hanke, F.; Havu, P.; Havu, V.; Ren, X.; Reuter, K.; Scheffler, M. Ab Initio Molecular Simulations with Numeric Atom-Centered Orbitals. *Comput. Phys. Commun.* **2009**, *180*, 2175–2196. https://doi.org/10.1016/j.cpc.2009.06.022.

(13) Tkatchenko, A.; Scheffler, M. Accurate Molecular van Der Waals Interactions from Ground-State Electron Density and Free-Atom Reference Data. *Phys. Rev. Lett.* **2009**, *102*, 073005. https://doi.org/10.1103/PhysRevLett.102.073005.

(14) Chemcraft - Graphical Software for Visualization of Quantum Chemistry Computations. Version 1.8, Build 682. https://www.Chemcraftprog.Com.

(15) Momma, K.; Izumi, F. *VESTA 3* for Three-Dimensional Visualization of Crystal, Volumetric and Morphology Data. *J. Appl. Crystallogr.* **2011**, *44*, 1272–1276. https://doi.org/10.1107/S0021889811038970.
